# Putatively cancer-specific exon–exon junctions are shared across patients and present in developmental and other non-cancer cells

**DOI:** 10.1093/narcan/zcaa001

**Published:** 2020-01-29

**Authors:** Julianne K David, Sean K Maden, Benjamin R Weeder, Reid F Thompson, Abhinav Nellore

**Affiliations:** Computational Biology Program, Oregon Health & Science University, Portland, OR 97239, USA; Department of Biomedical Engineering, Oregon Health & Science University, Portland, OR 97239, USA; Computational Biology Program, Oregon Health & Science University, Portland, OR 97239, USA; Department of Biomedical Engineering, Oregon Health & Science University, Portland, OR 97239, USA; Computational Biology Program, Oregon Health & Science University, Portland, OR 97239, USA; Department of Biomedical Engineering, Oregon Health & Science University, Portland, OR 97239, USA; Computational Biology Program, Oregon Health & Science University, Portland, OR 97239, USA; Department of Biomedical Engineering, Oregon Health & Science University, Portland, OR 97239, USA; Department of Radiation Medicine, Oregon Health & Science University, Portland, OR 97239, USA; Portland VA Research Foundation, Portland, OR 97239, USA; Department of Medical Informatics and Clinical Epidemiology, Oregon Health & Science University, Portland, OR 97239, USA; Division of Hospital and Specialty Medicine, VA Portland Healthcare System, Portland, OR 97239, USA; Cancer Early Detection Advanced Research Center, Oregon Health & Science University, Portland, OR 97239, USA; Computational Biology Program, Oregon Health & Science University, Portland, OR 97239, USA; Department of Biomedical Engineering, Oregon Health & Science University, Portland, OR 97239, USA; Department of Surgery, Oregon Health & Science University, Portland, OR 97239, USA

## Abstract

This study probes the distribution of putatively cancer-specific junctions across a broad set of publicly available non-cancer human RNA sequencing (RNA-seq) datasets. We compared cancer and non-cancer RNA-seq data from The Cancer Genome Atlas (TCGA), the Genotype-Tissue Expression (GTEx) Project and the Sequence Read Archive. We found that (i) averaging across cancer types, 80.6% of exon–exon junctions thought to be cancer-specific based on comparison with tissue-matched samples (*σ* = 13.0%) are in fact present in other adult non-cancer tissues throughout the body; (ii) 30.8% of junctions not present in any GTEx or TCGA normal tissues are shared by multiple samples within at least one cancer type cohort, and 87.4% of these distinguish between different cancer types; and (iii) many of these junctions not found in GTEx or TCGA normal tissues (15.4% on average, *σ* = 2.4%) are also found in embryological and other developmentally associated cells. These findings refine the meaning of RNA splicing event novelty, particularly with respect to the human neoepitope repertoire. Ultimately, cancer-specific exon–exon junctions may have a substantial causal relationship with the biology of disease.

## INTRODUCTION

Aberrant RNA splicing is increasingly recognized as a feature of malignancy (1–5), potentially driving cancer progression (4) and with potential prognostic significance across many cancer types, including non-small cell lung cancer, ovarian cancer, breast cancer, colorectal cancer, uveal melanoma and glioblastoma (6–11). Due to its potential for generating novel peptide sequences, aberrant RNA splicing is also interesting as a potential source of neoantigens for cancer immunotherapy targeting (12). For instance, retained intronic sequences can give rise to numerous potential antigens among patients with melanoma, although they are not a significant predictor of cancer immunotherapy response (13), and a patient-specific neoantigen arising from a gene fusion has been shown to lead to complete response from immune checkpoint blockade (14). Novel cancer-specific exon–exon junctions have also been shown to be a source of peptide antigens (15), and represent compelling potential targets for personalized anticancer vaccines (16).

However, the ability of the adaptive immune system to target a given antigen as ‘foreign’ depends on a complex prior tolerogenic education, and in particular on whether or not a given antigen has been previously ‘seen’ by the immune system in a healthy context (17). Therefore, prediction of cancer-specific antigens depends explicitly on their sequence novelty, and thus requires a comparison with non-cancer cells.

Choosing a ‘normal’ tissue standard for comparison is difficult in the context of RNA sequencing (RNA-seq) data analysis, given the presence of alternative splicing throughout normal and cancerous biological processes (2,18,19). Previously, cancer-specific aberrant splicing has been detected by comparing tumor RNA-seq data against a single reference annotation (20) or a limited ‘panel of normals’ (13). A TCGA network paper (15) used the large publicly available datasets of The Cancer Genome Atlas (TCGA) (21) and the Genotype-Tissue Expression (GTEx) Project (22) to identify and validate thousands of novel splicing events, including exon–exon junctions present in a specific TCGA cancer type but not in the corresponding normal adult tissue in GTEx. This study also predicted alternative splicing neoepitopes via this comparison, and validated several of these neoepitopes shared between multiple patients with the intracellular proteomics data available for select ovarian and breast cancer TCGA donors in the Clinical Proteomic Tumor Analysis Consortium dataset (15). More recently, another study has leveraged TCGA and GTEx, as well as cell line data, to discover and validate neoepitopes derived from alternative splicing (23).

Here, we propose that the comparison of cancer junctions with only matched normal GTEx tissue data allows a significant number of junctions to be erroneously identified as cancer-specific, and that GTEx provides neither an appropriately specific nor a fully comprehensive standard for normal splicing comparison. We investigate the sharedness of cancer junctions within and across cancer type cohorts, and their presence across multiple normal cell and tissue types, including cohorts representing diverse developmental stages and potential cell types of cancer origin.

## MATERIALS AND METHODS

### Data download

Previously called exon–exon junction data including phenotype table, bed and coverage files for both TCGA and GTEx v6 were downloaded from the recount2 service at https://jhubiostatistics.shinyapps.io/recount (24). These data were previously extracted (25) from RNA-seq experiments encompassing 10 549 tumor samples across 33 TCGA cancer types, 788 paired normal samples across 25 TCGA cancer types and 9555 normal samples across 30 GTEx tissue types (Supplementary Table S1). recount2 used Rail-RNA (26) to align RNA-seq samples, and all command-line parameters affecting alignment are referenced in supplementary information from the recount2 paper (25). The metaSRA (27) web query form at http://metasra.biostat.wisc.edu/ (a tool for identifying SRA samples of interest) was queried for experiment accession numbers for (i) non-cancer cell and tissue type samples (see Supplementary Table S1 for cancer-matched samples and Supplementary Table S3 for non-cancer samples, and ‘Comparison with SRA tissue and cell types’ section for a description of how these samples were chosen) and (ii) TCGA-matched cancer types (see Supplementary Table S1). For the non-cancer samples, the term ‘cancer’ was explicitly added as an excluded ontology term in the query, and the resulting files were filtered to remove any samples with ‘tumor’ in the sample_name field. The resulting accession numbers represent 12 231 human samples from the SRA, specifically 10 827 samples from 33 normal tissue and cell types and 1404 samples from 14 cancer types (Supplementary Tables S1 and S3). These accession numbers were queried against the Snaptron junction database using the query snaptron tool (for interfacing with uniformly extracted recount2 junctions) (25,28). This query yielded junctions also previously extracted by recount2 with the same pipeline used for the GTEx and TCGA samples for the tissue and cell types of interest (25), which were subsequently downloaded. TCGA tumor mutational burden (TMB) data (file mutation-load-updated.txt) were downloaded from https://gdc.cancer.gov/about-data/publications/PanCan-CellOfOrigin (29). Patient somatic mutation calls were downloaded from the GDAC firehose (30), while a list of human splicing-associated gene mutations (keyword search ‘mRNA splicing [KW-0508]’) was downloaded from the UniProt database (31). Two lists of cancer-associated genes were downloaded: the COSMIC Cancer Gene Census cancer gene list from https://cancer.sanger.ac.uk/census (32) and the OncoKB cancer gene list from https://oncokb.org/cancerGenes (33). The GENCODE v.28 comprehensive gene annotation file (gencode.v28.annotation.gtf, the noted "main annotation file") was downloaded from https://www.gencodegenes.org/human/release_28.html (34).

### Indexing of GTEx and TCGA junctions

The GENCODE gene transfer format (.gtf) file (34) was parsed to collect full coordinates and left and right splice sites of junctions from annotated transcripts and a searchable tree of protein-coding gene boundaries. The GTEx phenotype file was parsed to collect tissue-of-origin information and donor gender; bone marrow samples derived from leukemia cell line cells were eliminated. The TCGA phenotype file was parsed to collect information on cancer type, cancer stage at diagnosis, patient gender, vital status and sample type (primary tumor, matched normal sample, recurrent tumor or metastatic tumor). Cancer subtype classifications were collected for five cancer types beyond their TCGA designations (Figure 2B, Supplementary Table S1): cervical squamous cell carcinoma and endocervical adenocarcinoma was separated into cervical squamous cell carcinoma, endocervical adenocarcinoma and cervical adenosquamous; esophageal carcinoma was separated into esophagus adenocarcinoma and esophagus squamous cell carcinoma; brain lower grade glioma was separated into astrocytoma, oligoastrocytoma and oligodendroglioma; sarcoma was separated into leiomyosarcoma, myxofibrosarcoma, malignant peripheral nerve sheath tumors, desmoid tumors, dedifferentiated liposarcoma, synovial sarcoma and undifferentiated pleomorphic sarcoma; and pheochromocytoma and paraganglioma were separated. A new SQLite3 database was created to index all GTEx and TCGA junctions, with linked tables containing (i) sample IDs and associated junction IDs; (ii) sample IDs and phenotype information for each sample; and (iii) junction IDs and junction information, including 0-based closed junction coordinates, GENCODE annotation status and location within protein-coding gene boundaries. SQL indexes were created on junction ID and sample ID columns for fast and flexible querying.

### Selection of cancer-specific junction filters

For all analyses we apply a light filter, requiring a junction to have at least a two-read coverage across GTEx, TCGA and the selected cancer and non-cancer SRA samples, to exclude false positive junctions but allow for the existence of splicing noise; we do not require a minimum read count per sample. To characterize junction novelty in cancer with respect to normal cells, we defined a hierarchical filter that specifies inclusion and exclusion of junctions in different RNA-seq datasets (Table 1). In order from most to least permissive, these filters are (i) junctions not found in tissue-matched GTEx or TCGA normal samples; (ii) junctions not found in any GTEx or TCGA normal (‘core normal’) samples; and (iii) junctions not found in any core normal samples or in selected SRA tissue and cell type non-cancer samples. For our analyses, we do not explicitly filter on whether a junction is annotated in GENCODE. We do not set a limit on presence in the core normal sample cohorts: any junction present at any coverage level in only one sample is counted as ‘in’ these cohorts. This yields a more stringent filter on normality than that used by the TCGA splicing paper, which uses the term ‘neojunctions’ to refer to junctions not found in tissue-matched GTEx or TCGA normal samples, with a 10-read coverage requirement in TCGA, and allowing through the filter lowly expressed junctions in GTEx tissue-matched samples (15).

**Table 1.** Junction novelty specification

Junction novelty stage	Definition
0	All junctions
1+	Junctions not found in tissue-matched GTEx or TCGA normal samples
2+	Junctions not found in any GTEx or TCGA normal (‘core normal’) samples
3+	Junctions not found in any core normal samples or in selected SRA tissue and cell type non-cancer samples

### Extraction and analysis of cancer-specific junctions

We queried the junction database to extract junctions of interest, specifically (i) all junctions for all tumor samples of each cancer type and (ii) all junctions not present in any core normal samples for each cancer type cohort, with their cohort prevalence levels. All junctions are presented in a 0-based closed coordinate system. We also identified a set of ‘shared junctions’ for every cancer type, defined as up to 200 most highly recurring junctions that occur in at least 1% of the cancer type samples and are not found in any core normal samples. Protein-coding region presence was determined for all junctions, with location assessment as follows: the junction is categorized as protein-coding if it is present in a protein-coding gene region (with at least one junction splice site within the gene boundaries) and antisense if it is present on the reverse strand of a protein-coding gene region, based on gene regions described in GENCODE v.28 (34). Cancer-associated genes were collected from the OncoKB and the COSMIC Cancer Gene Census; any gene listed in one or both lists was categorized as a cancer-associated gene. Any junction assigned to a protein-coding gene region corresponding to one of these genes was categorized as associated with cancer-relevant loci.

For comparison between cancer sample junctions found and not found in core normal samples, we performed a Kruskal–Wallis *H*-test to determine the significance of the decreased sharedness levels, since the junction prevalence data are not normally distributed and there are many fewer cancer-specific junctions than junctions found in core normal samples.

### Comparison with SRA tissue and cell types

Non-cancer sample types from the SRA were chosen via manual curation informed by a clustering of junctions according to ontology term prevalence, with commonly occurring terms that do not meaningfully distinguish junctions eliminated. The selected sample types in Supplementary Table S3 comprise all non-cancer data from the SRA analyzed. All junctions for samples associated with these cell and tissue types but not with ‘cancer’ were downloaded via Snaptron, translated to a 0-based closed coordinate system and compared with those found in TCGA cancer samples. Junctions present in a TCGA cancer type cohort and SRA samples from a specific assigned category determined set assignments, which were used for subsequent data analysis. To exclude false positive junctions but allow for the existence of splicing noise, only junctions with at least two reads across GTEx, TCGA and the selected cancer and non-cancer SRA samples are considered true junctions. All SRA junctions not found in TCGA cancer samples were ignored. For the supplementary two-sample minimum filter analysis, we retained all junctions that are present in only one SRA sample, but required at least two samples across the broad SRA category (adult, developmental or stem cell) for inclusion in that set. (For developmental subsets, only one sample within a subset category was required, as long as the two-sample criterion across the full developmental category was met.)

For comparison between TCGA cancer sample junctions not found in core normal samples with SRA junctions from matched cancer type samples, we performed a Kruskal–Wallis *H*-test to determine the significance of the increased sharedness levels, since the junction prevalence data are not normally distributed and the difference in junction counts between the two cohorts (TCGA junctions in or not in the SRA-matched cohort) is large.

### Comparison of junction burden and TMB

Silent and non-silent mutations per Mb per patient were added to give a total TMB per patient. Junctions considered for the ‘junction burden’ calculation were all tumor sample junctions not found in core normal samples. The total junction count per patient was divided by the mapped read count of the sample divided by 10 000 (scaling to ‘per Mb’ with the assumption of 100-bp reads) to give the final junction burden. A linear regression was performed on the junction burden versus TMB across all TCGA tumor samples.

### Splicing factor mutation analysis

Patient somatic mutation call files were downloaded from the GDAC firehose (http://gdac.broadinstitute.org/) (30). While we note the potential importance of mutations in non-coding sequences, we confined our attention exclusively to non-synonymous mutations. Patients were classified based on two different separation criteria: (i) a *de novo* analysis of whether or not they had at least one mutation in a gene that codes for a protein annotated as involved in mRNA splicing, based on the UniProt protein annotation database; and (ii) whether or not they had at least one mutation in a gene previously identified as sQTL associated (U2AF1, SF3B1, TADA1, PPP2R1A and/or IDH1) in the TCGA cohort by the TCGA splicing paper (15). For each cancer type, and each stratification method, the number of cancer-specific junctions per patient was compared for patients with and without at least one mutation in the defined set (Supplementary Figure S1F and S1G). Differences in the number of novel junctions across cancer types and stratification groups were assessed via two-way ANOVA with a Benjamini–Hochberg *P*-value correction.

In addition to comparing the levels of cancer-specific junctions between patients with and without splicing-associated mutations, we also compared junction sharedness based on the same two stratification criteria used earlier. For each cancer type, all junctions identified in two or more patients were selected. For each, the number of junction occurrences in patients with mutations in splicing-associated genes was calculated and compared to the overall number of occurrences in the corresponding cancer cohort, using a Fisher's exact test (Supplementary Figure S1H and S1I).

### Survival analysis for ovarian cancer patients with target antisense MSLN junction

All TCGA ovarian patients with data in our TCGA phenotype file in columns ‘xml days to last followup’ or ‘gdc cases.diagnoses.days to death’ were included in the survival analysis. The survival curve was plotted for the second column, with dropout patients with no days-to-death data censored at days to last follow-up.

## RESULTS

### Cancers harbor many novel shared exon–exon junctions not present in adult non-cancer tissues or cells

**Figure 1.**
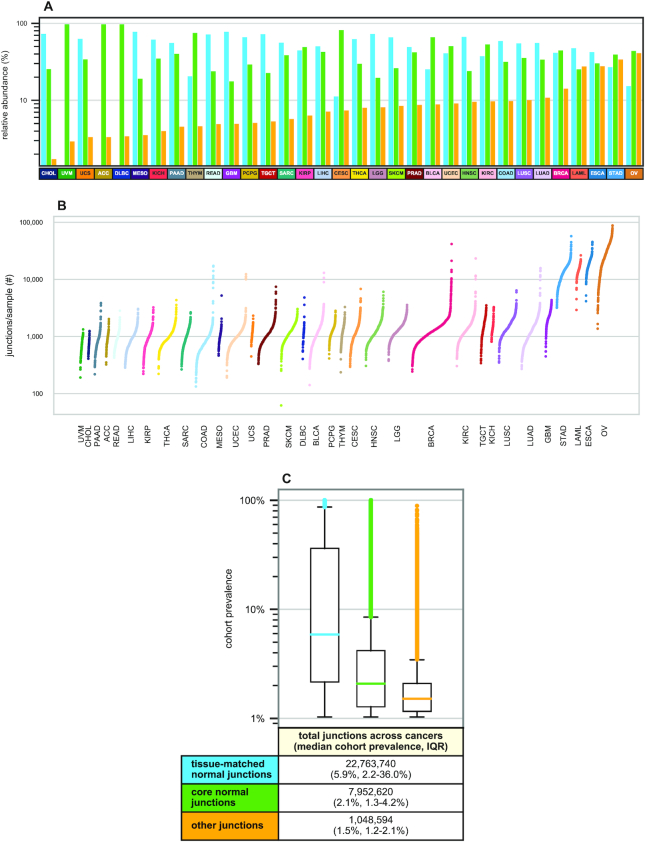
Distribution of exon–exon junctions across and within TCGA cancer cohorts. (**A**) Log-scale bar charts describing the percentage of all junctions of a given cancer type cohort present in three subcohorts. Blue (left) bars give the percentage of cohort junctions found in GTEx or TCGA tissue-matched normal samples (Supplementary Table S1), green (center) bars give the percentage of the remaining junctions that are found in other core normals and yellow (right) bars give the percentage of cohort junctions not found in any core normals; cancer types are ordered by relative abundance of junctions in this last set. Cancer types with no blue (left) bar have no tissue-matched normal samples (Supplementary Table S1). (**B**) Log-scale sorted strip plots representing the number of non-core normal junctions per sample for each of 33 TCGA cancer types. Each point represents a single TCGA tumor sample and the width of each strip is proportional to the size of the cancer type cohort (15). Supplementary Figure S1B shows analogous data with additional filters applied. (**C**) Log-scale box plots representing the prevalences within each cancer type cohort of junctions occurring in at least 1% of cancer type samples, summarized across all TCGA cancer types. Junction prevalences are shown in blue (left) for those found in GTEx or TCGA tissue-matched normal samples (Supplementary Table S1), junctions not present in tissue-matched normals but found in other core normals are shown in green (center) and junctions not found in any core normals are shown in yellow (right). Note that any junction found in multiple cancer types is represented by multiple data points, one for each cancer type in which it is found. A detailed breakdown by TCGA cancer type is available in Supplementary Figure S1E.

While cancer-specific exon–exon junctions identified using tissue-matched normal samples have the potential to give rise to neoantigens (15), we reasoned that they could be expressed in other normal tissues due to variability in patterns of transcription and alternative splicing among different tissues (35). In such cases, these junctions might not yield bona fide neoantigens due to the prior tolerogenic education of the immune system. We therefore re-evaluated the incidence of cancer-specific junctions using RNA-seq data from TCGA and the large compendium of adult tissues from GTEx. We found that on average, across cancer types, 80.6% of junctions potentially thought to be cancer-specific based on comparison only with tissue-matched samples (*σ* = 13.0%) are in fact present in other adult non-cancer tissue and cell types throughout the body. Across cancer types, an average of 90.2% of all junctions found in cancer samples (*σ* = 9.1%) are also present in one or more adult normal samples from GTEx or TCGA (‘core normals’, Figure 1A). The overall number of these novel junctions varies both within and across different cancer types, with ovarian carcinoma and uveal melanoma having the highest and lowest average number of junctions per sample, respectively (Figure 1B, Supplementary Table S1), and is independent of TMB (Supplementary Figure S1A). The set of junctions defined as ‘novel’ is highly sensitive to the filtering criteria used (see Supplementary Figure S1B, Supplementary Table S2 and ‘Selection of cancer-specific junction filters’ section). We are interested in junctions that are widely expressed across samples, and for this analysis we sought to optimize sensitivity and specificity to detect shared cancer-specific junctions. High prevalence across a cancer type cohort provides strong support for the existence of junctions, despite low coverage of these junctions within any individual sample; we require a minimum of two reads across all studies, but do not set a lower bound on sample coverage. Going forward, we use strict lack of occurrence of a junction in core normals as our baseline definition of cancer specificity, where even a single read in the target ‘normal’ set eliminates a junction from the cancer-specific designation (see ‘Selection of cancer-specific junction filters’ section).

We next assessed the extent to which a given junction not found in core normals might be shared among multiple samples of the same cancer type. We observed that over half (52.8%) of these junctions are confined to individual samples, although a small but significant subset (0.41%) is shared across at least 5% of samples in at least one cancer type cohort (Supplementary Figure S1C). We also noted that 40.6% of novel junctions are shared between multiple cancer types, with a total of 1609 junctions present in at least 5% of samples each across two or more TCGA cancer cohorts (Supplementary Figure S1D). Sharedness was significantly higher among junctions that were also present in normal tissues (Figure 1C, Supplementary Figure S1E). We observed that the number of junctions not found in core normals per patient was comparable for patients with and without splicing factor-associated mutations across all cancer types, with the exception of breast adenocarcinoma (Supplementary Figure S1F and S1G). We also observed that splicing-associated mutations had minimal effect on the sharedness within a cancer type cohort of junctions not found in core normals (Supplementary Figure S1H and S1I).

We finally assessed whether these junctions were also shared among independent cancer cohorts, using publicly available RNA-seq data in the Sequence Read Archive (SRA) (36). Many TCGA cancer junctions not found in core normals were found to occur in cancer type-matched SRA samples: 11 of 14 cancer types had more than 50 junctions in common between the matched cohorts. Moreover, we found that junctions also present in matched SRA cancer cohorts were associated with significantly higher levels of sharedness in the TCGA cohort (*H* statistic = 3.85–2803 and *P* = <0.0001–0.0495; Supplementary Figure S1J).

### Shared novel junctions in cancer distinguish cancer identity and subtype

**Figure 2.**
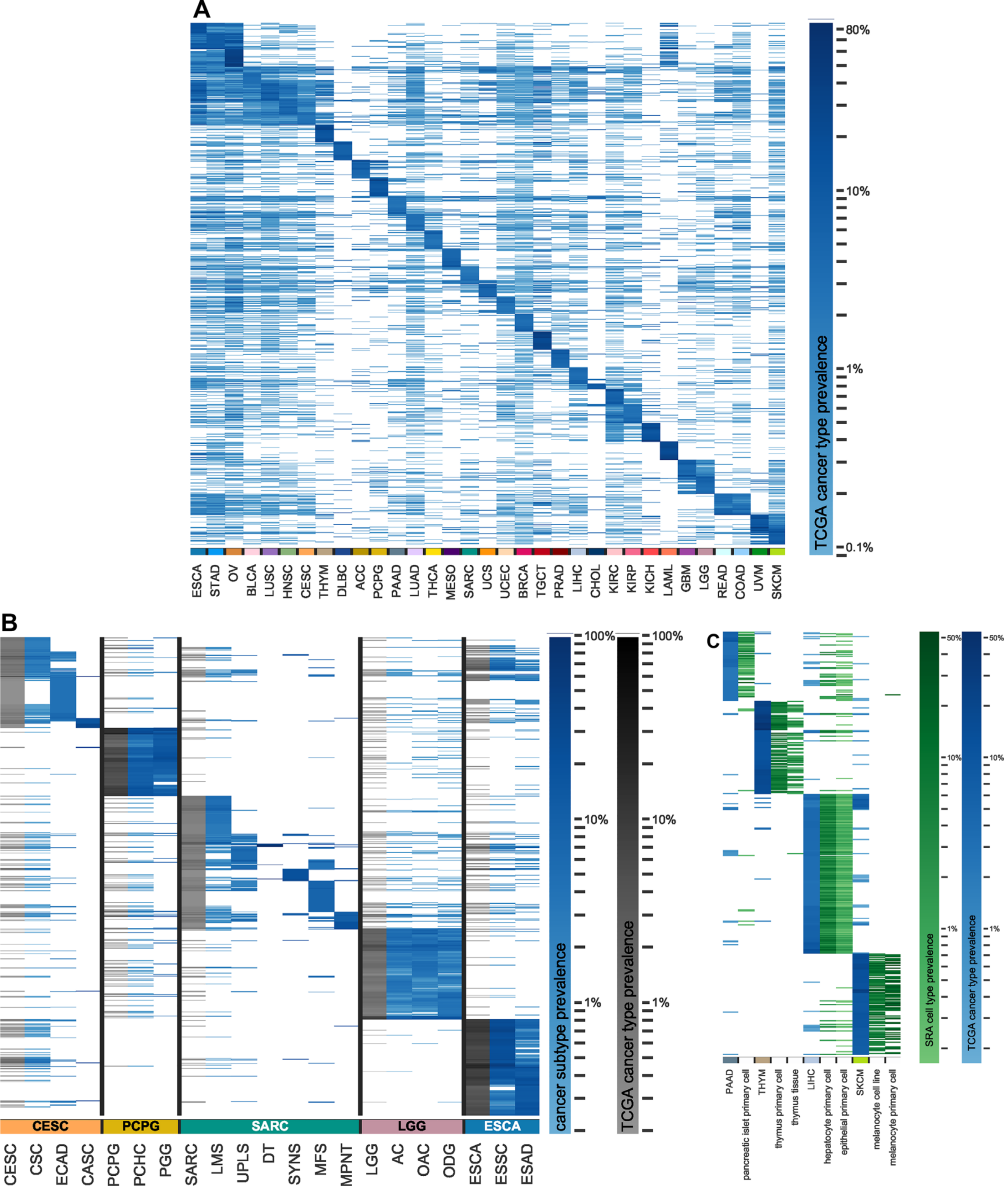
Clustering by cohort prevalence of shared novel junctions not found in core normal samples. (**A**) Heatmap showing junction prevalences across every TCGA cohort for each cancer type's top 200 shared junctions that are at least 1% prevalent in that cancer type and are not found in any core normal samples. (**B**) Heatmap showing shared junction prevalences across selected TCGA cancer types and their assigned histological subtypes for each subtype's top 200 shared junctions that are at least 1% prevalent in that subtype and are not found in any core normal samples. See Supplementary Table S1 for TCGA subtype abbreviations. (**C**) Heatmap showing shared junction prevalences across selected TCGA cancer types and a set of their matched SRA tissue and cell types of origin, for each cancer type's top 200 shared junctions that are at least 1% prevalent in that cancer cohort and are not found in any core normal samples.

We hypothesized that a high level of exon–exon junction sharedness across samples is likely to be reflective of underlying conserved biological processes (e.g. among normal tissues). We therefore investigated the sharedness of novel junctions present in different cancer types. Interestingly, these novel junctions can readily distinguish disparate cancer types and show similarities among cancer types with shared biology, such as cutaneous and uveal melanomas (Figure 2A). These novel junctions also reflect shared biology among additional cancer types with similar anatomic origins: colon and rectal adenocarcinoma, clear cell, chromophobe and papillary renal cell carcinomas, low- and high-grade gliomas, and stomach and esophageal adenocarcinomas (Figure 2A). Shared junctions from several cancer types also demonstrate similarities by histological subtype despite their differing anatomical origins, for instance squamous cell carcinomas of the lung, cervix, and head and neck (Figure 2A, Supplementary Figure S2A), consistent with previously published work (37). Moreover, shared novel junctions are readily able to distinguish distinct histological subtypes of sarcoma and cervical cancer, among other diseases (Figure 2B). Using non-cancer cell types from the SRA, we found that ‘novel’ junctions from cancers arising from cell and tissue types poorly represented in GTEx normal tissue samples (e.g. melanocytes), or not present in GTEx at all (e.g. thymus tissue), can be found in many samples of the corresponding cell or tissue types of origin (Figure 2C, Supplementary Table S1). Sample-to-sample comparisons of all junctions from these rare-cell type cancers also show more similarity with cell type-matched normal samples from the SRA than with bulk tissue from GTEx (Supplementary Figure S2B).

### Novel junctions in cancer are found among developmental and known cancer-related pathways

**Figure 3.**
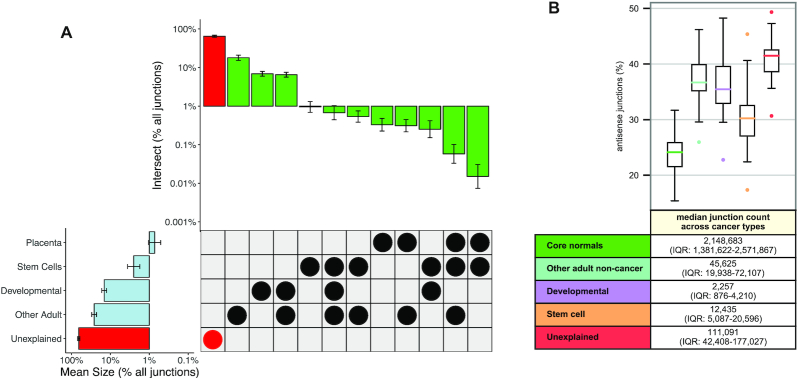
Junction set assignments and antisense junction prevalence in additional normal tissue and cell type categories from the SRA, across cancers. (**A**) Upset-style plot with bar plots showing junction abundances across major sets (left) and set overlaps (top) across 33 cancers (error bars). Shown junctions are absent from all core normals. Unexplained junctions (red highlights) comprise junctions not present in any set categories studied (see also expanded set assignments in Supplementary Figure S3A). The developmental set comprises human development-related junctions not present in the category placenta. Scale is log_10_ of percent of junctions not found in core normals, calculated for each cancer. (**B**) Box plots showing, for each TCGA cancer type, the percent of junctions that are antisense for (green) junctions found in core normals, (aqua) junctions not found in core normals but found in other selected non-cancer adult tissue and cell samples from the SRA, (lavender) junctions not found in core normals or SRA non-cancer adult samples but found in selected developmental samples on the SRA, (apricot) junctions not found in core normals or SRA non-cancer adult samples but found in selected stem cell samples on the SRA and (red) junctions not found in core normals or selected non-cancer adult, developmental or stem cell samples from the SRA. Each point represents the percent of junctions from one cancer type in the given category (e.g. developmental) that are antisense. The table shows the median and interquartile range of the number of junctions in that category across all TCGA cancer types.

As many cancers are thought to recapitulate normal developmental pathways (38–40), we further hypothesized that a subset of cancer-specific junctions may reflect embryological and developmental splicing patterns. We therefore compared cancer junctions not found in core normals with those from SRA samples pertaining to zygotic, placental, embryological and fetal developmental processes: on average, per cancer type, 15.4% of these cancer junctions (*σ* = 2.4%) occur in SRA developmental cell or tissue samples. We also considered samples from SRA normal stem cell samples and from selected SRA normal adult tissue and cell types: on average, per cancer type, 2.7% (*σ* = 1.4%) and 26.5% (*σ* = 3.3%) of cancer junctions not found in core normals occur in stem cell and selected adult tissues, respectively (Figure 3A, Supplementary Figure S3A). Furthermore, many of the junctions found in SRA developmental, stem cell and selected adult tissues are highly prevalent shared junctions (Supplementary Figure S2A). The remaining significant majority of these cancer junctions not found in core normals were also not found in any non-cancer SRA tissue or cell type studied [64.9% on average per cancer type cohort (*σ* = 4.0%), Figure 3A, Supplementary Figure S3A]. Many of these novel ‘unexplained’ junctions still exhibit high levels of sharedness both within (Supplementary Figure S3B and S3C) and between (Supplementary Figure S3D) different cancer types. At the upper end, 16 of these shared junctions were found in more than 10% of samples in each of two or more cancer types (Supplementary Table S4).

We note that the liberal set inclusion criterion we employed may reduce our ability to identify robust cancer-specific biology among unexplained junctions. For instance, the well-described deletion causing a splicing of exons 1 and 8 (*EGFRvIII*) occurs in 29.4% of TCGA patients with glioblastoma multiforme (GBM) and in non-core normals, but is also present in a single read from a single human epithelial cell line sample on SRA, and therefore is classified not as an unexplained cancer-specific junction but as ‘adult non-cancer’. However, this set inclusion condition does allow for the identification of some cancer-specific biology of interest. For instance, rarer alternative *EGFR* splicing events were detected in the unexplained set, such as *EGFRvIII* with an alternate exon 1 joined to exon 8 (chr7:55161631–55172981), detected in two patients with GBM and one patient with low-grade glioma, the same alternate exon 1 joined with two alternate exon 16s (chr7:55161631–55168521 and chr7:55161631–55170305, detected in one and two GBM patients, respectively) and the same alternate exon 1 joined with exon 20 (chr7:55161631–55191717) in two GBM patients. An alternative filtering approach that instead requires two samples per SRA category to define junction set membership yields a greater number of unexplained junctions (Supplementary Table S2, Figure S3E and S3F).

We observed a number of unexplained junctions shared by unusually large proportions of ovarian cancer samples in TCGA, including one cancer-specific junction (chr16:766903–768491 on the minus strand) present in the highest proportion of samples in any TCGA cohort (81.3%, or 350 of 430 ovarian cancer samples). This junction occurs in an antisense transcript of *MSLN*, which codes for a protein known to bind to the well-known ovarian cancer biomarker *MUC16* (CA125) (41,42). The functional consequence of this junction is unknown, but it does not appear to affect overall survival (Supplementary Figure S3G). Another unexplained junction (chr19:8865972–8876532 on the minus strand) is in the *MUC16* region itself and is present in 42.8%, or 184 of 430 ovarian cancer samples. In all, we identified 34 cancer-specific junctions present in >40% of ovarian cancer samples. We further identified several novel pan-cancer splice variants (chr16:11851406 with chr16:11820297, chr16:11821755 and chr16:11828391, each present across up to eight different cancers) in *RSL1D1* and its neighboring *BCAR4*, a long non-coding RNA known to promote breast cancer progression (43,44).

Among all otherwise unexplained junctions, an average of 4.78% (*σ* = 0.48%) across cancer types are associated with known cancer-predisposing or cancer-relevant loci. Further, an elevated proportion of otherwise unexplained junctions (on average, 40.9%, *σ* = 3.8%) occur in likely antisense transcripts and may therefore be of reduced interest as candidate neoantigens, but sustained interest in terms of cancer biology (Figure 3B, Supplementary Table S5). Finally, we show that 20 genes not previously known to be cancer-associated each contain at least 25 novel, unexplained junctions present in at least 5% of samples of at least one cancer type (Supplementary Table S6).

## DISCUSSION

Previous studies have established the importance of alternative and aberrant splicing in cancer prognosis (6–11) and have begun to explore its potential relevance in cancer immunotherapy (15,23,45). In this study, we explore ‘novel’ exon–exon junctions used among cancers with respect to a broad collection of normal tissue and cell types. This is the largest such study to date integrating RNA-seq data from 10 549 tumor samples across 33 TCGA cancer types, 788 paired normal samples across 25 TCGA cancer types, 9555 normal samples across 30 GTEx tissue types and 12 231 human samples from the SRA (10 827 samples from 33 normal tissue and cell types and 1404 samples from 14 cancer types) (Supplementary Tables S1 and S3). To the best of our knowledge, this is also the first study to examine the novelty of cancer junctions from the perspective of immune tolerance, considering all adult normal tissue types as potential sources of tolerogenic peptides rather than only the closest matched normal tissues. Moreover, this is the first study to quantitatively interrogate the sharedness of novel exon–exon junctions both within and across cancer types, demonstrating that these junctions can distinguish some cancers and their subtypes. We finally demonstrate that there is no one-size-fits-all definition of ‘novel’ splicing, noting that purportedly cancer-specific junctions may in fact be present among, and perhaps biologically consistent with, a repertoire of embryological, developmentally associated and other cell types.

This study also has several limitations. We focus on the importance of exon–exon junctions as the predominant metric of alternative splicing, in particular on their presence or absence among different samples, but do not explore the potential for differences in gene dosage to drive differences in biology. Moreover, there are other sources of RNA variation (e.g. intron retention events (13) and RNA editing) that we do not explicitly study here, but which could be equally good sources of novel, cancer-specific protein sequence for immunotherapeutic and other applications. Importantly, there is substantial variability among analytical methods for identifying these exon–exon junctions. We note significant discordance between results of analyses of the same data using different junction filtering methods. While the same phenomena and general results appear to hold true independent of analytical technique, the identity and relative novelty of individual ‘cancer-specific’ junctions vary between our results and those previously published (15). We also acknowledge that GTEx and the SRA combined do not account for all sources of normal tissue(s) in the human body, and further acknowledge that the sample metadata used to search the SRA may be an imperfect surrogate for actual tissue/sample identities. Our assessment of embryological and developmentally associated junctions is also limited by a relatively small number of relevant RNA-seq samples available on the SRA. Our splicing factor mutation analysis was also limited by sample size and was confined exclusively to non-synonymous mutations. Finally, due to the short-read nature of these RNA-seq data, we make no attempt to predict putative neoepitopes from cancer-specific junctions as we cannot confidently recapitulate reading frame or broader sequence context from isolated exon–exon junctions, particularly without access to the biological specimens to perform junction-level experimental validation.

While cancer-specific exon–exon junctions may indeed be a source of neoepitopes, their sharedness across individuals and occurrence in cancer-relevant loci (e.g. *EGFR*, *MUC16*) are suggestive of underlying but as-of-yet unexplored biology. This sharedness does not appear to be related to variants in splicing factor or splicing-associated proteins, and is not wholly explained by recapitulation of embryological/developmental transcriptional profiles. As such, we see this work as opening a broad area of future research into the role and relevance of these novel recurring exon–exon junctions.

## DATA AVAILABILITY

All data are publicly available and accessible online as described in ‘Data download’ section. Python code and corresponding descriptors for the implementation of methods as described are publicly available on GitHub (https://github.com/JulianneDavid/shared-cancer-splicing).

## Supplementary Material

zcaa001_Supplemental_File

## Notes

**Disclaimer:** The contents do not represent the views of the United States Department of Veterans Affairs or the United States Government.
