# Supplementary material for: Putatively cancer-specific exon–exon junctions are shared across patients and present in developmental and other non-cancer cells
Source: NAR Cancer. 2020 Jan 29;2(1):zcaa001. doi: 10.1093/narcan/zcaa001 (PMC8209686; doi:10.1093/narcan/zcaa001)
Supplement: zcaa001_Supplemental_File [file narcancer_2_1_zcaa001_s1.pdf]

Supplemental Information

(A)

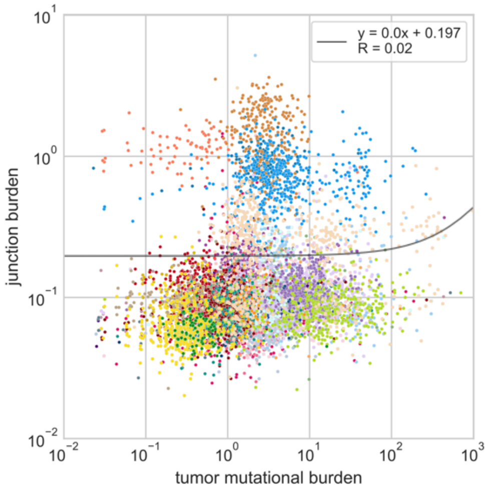

(B)

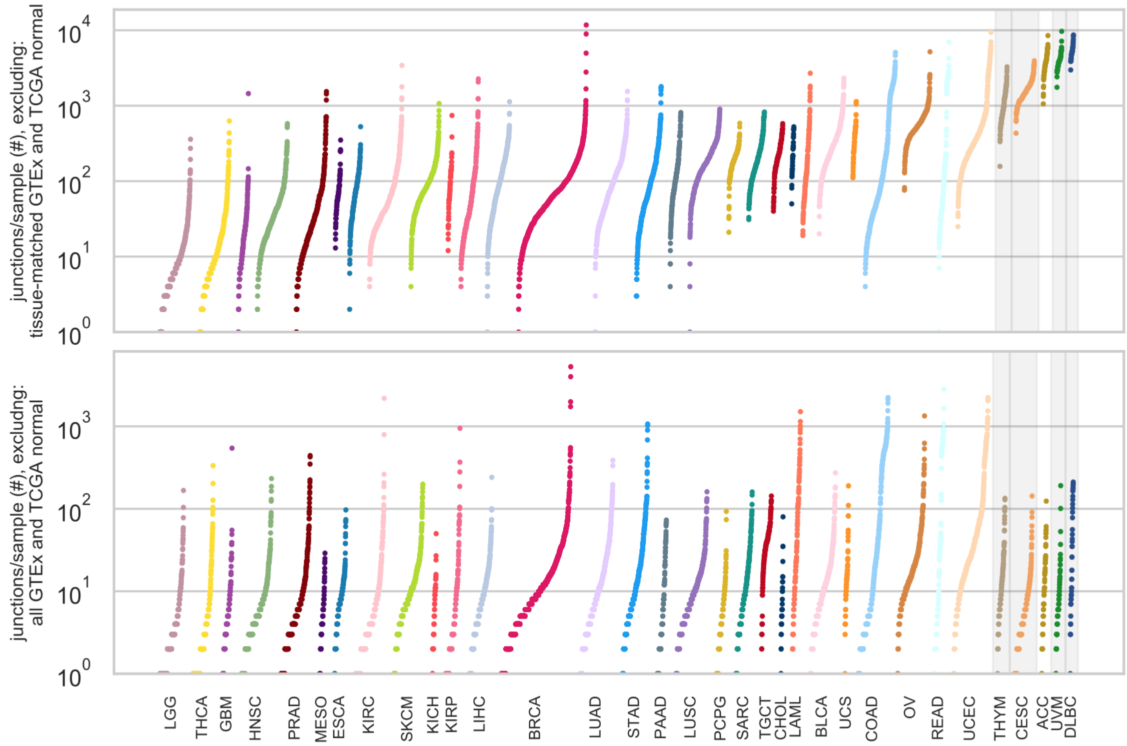

(C)

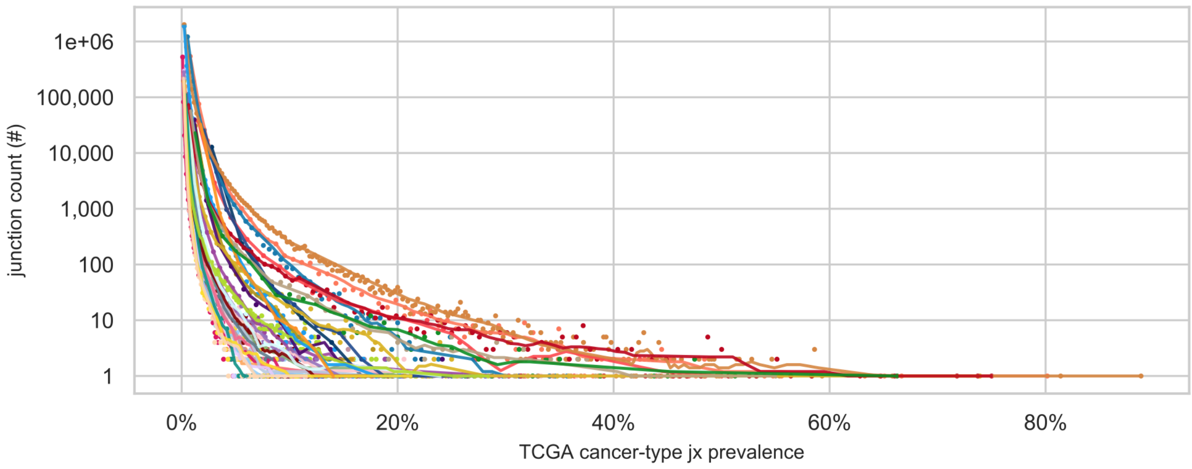

(D)

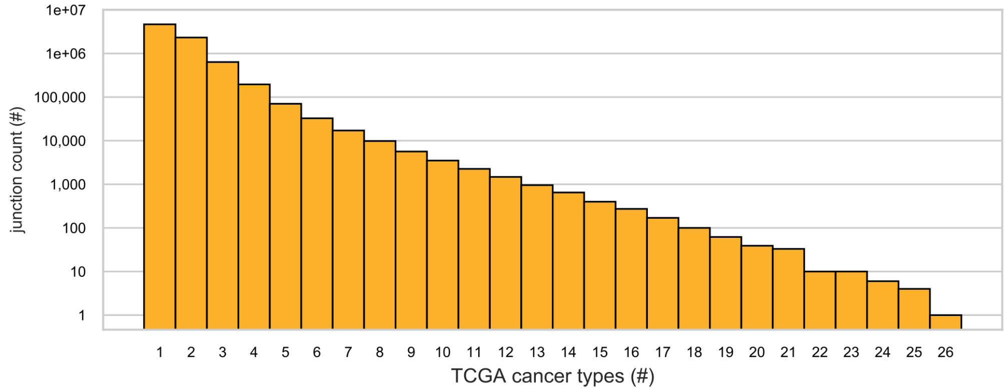

(E)

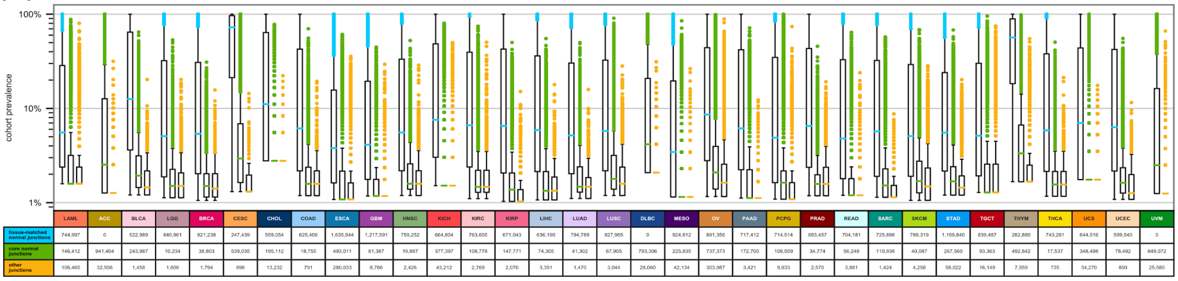

	LAML	ACC	BLCA	LGG	BRCA	CESC	CHOL	COAD	ESCA	GBM	HNSC	KICH	KIRC	KIRP	LIHC	LUAD
tissue-matched normal junctions	744,997	0	522,989	840,961	821,238	247,439	559,054	625,406	1,635,944	1,217,591	759,252	664,804	763,605	671,043	636,190	794,789
core normal junctions	146,412	941,404	243,987	10,234	38,803	539,035	195,112	18,755	493,011	61,387	19,887	377,397	108,778	147,771	74,305	41,302
other junctions	106,465	32,508	1,458	1,606	1,794	896	13,232	791	280,033	8,786	2,426	43,212	2,769	2,070	3,351	1,470

	LUSC	DLBC	MESO	OV	PAAD	PCPG	PRAD	READ	SARC	SKCM	STAD	TGCT	THYM	THCA	UCS	UCEC	UVM
tissue-matched normal junctions	827,965	0	924,812	891,356	717,412	714,514	693,457	704,181	725,898	789,319	1,159,840	839,487	282,880	743,261	644,516	599,540	0
core normal junctions	67,905	793,306	225,835	737,373	172,700	108,509	34,774	56,249	110,938	40,087	267,560	93,363	492,842	17,537	348,498	78,492	849,072
other junctions	3,044	28,060	42,134	303,987	3,421	9,833	2,570	3,881	1,424	4,258	58,022	16,149	7,559	735	34,270	800	25,580

(F)

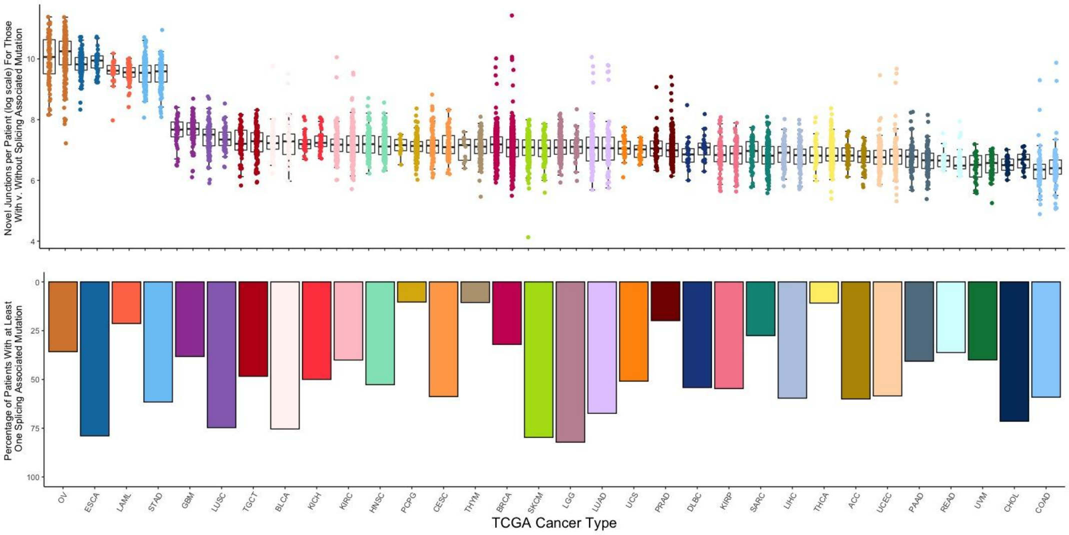

(G)

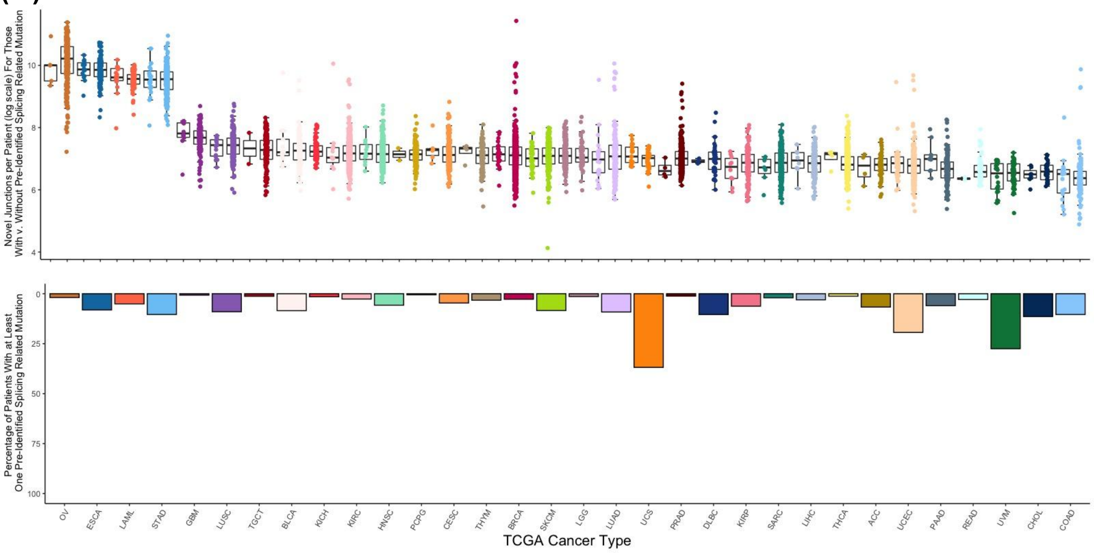

(H)

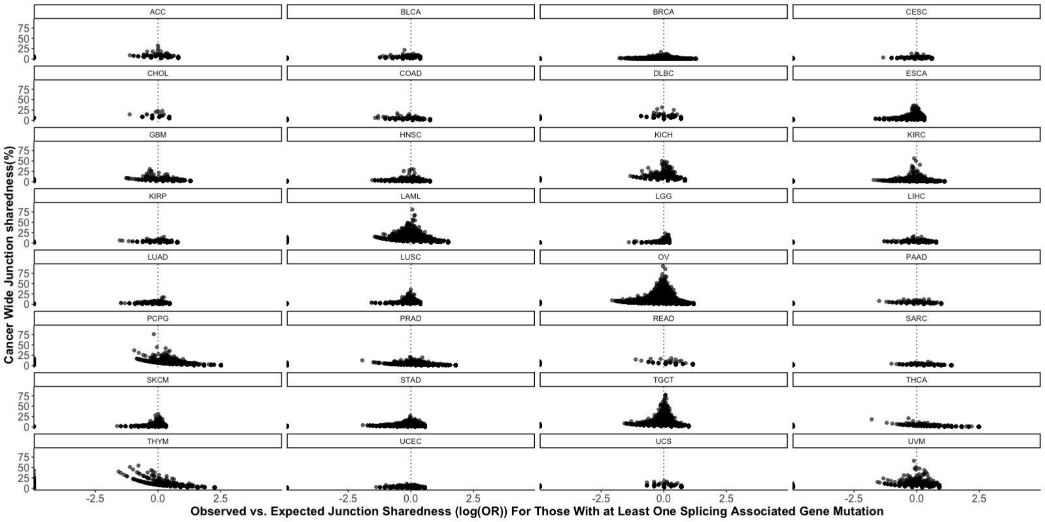

(I)

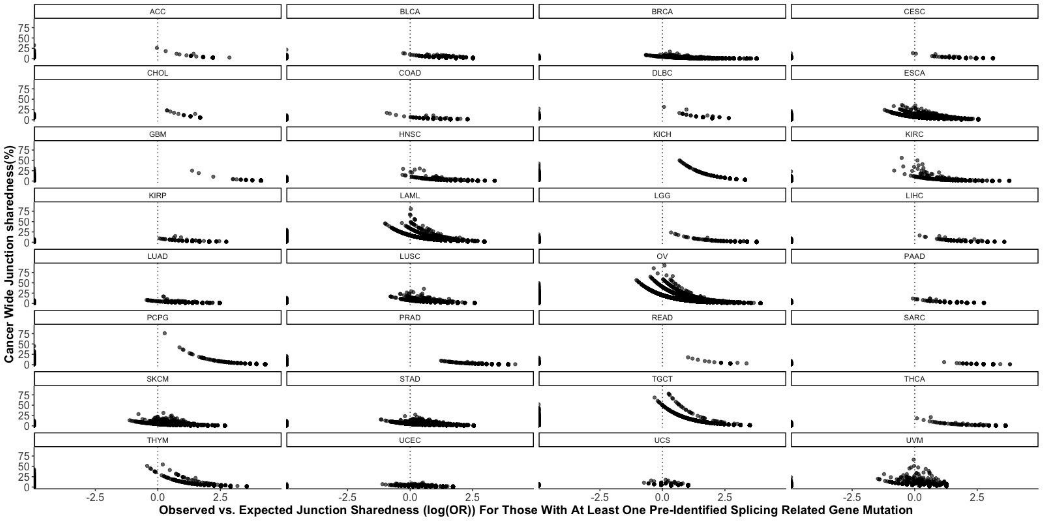

(J)

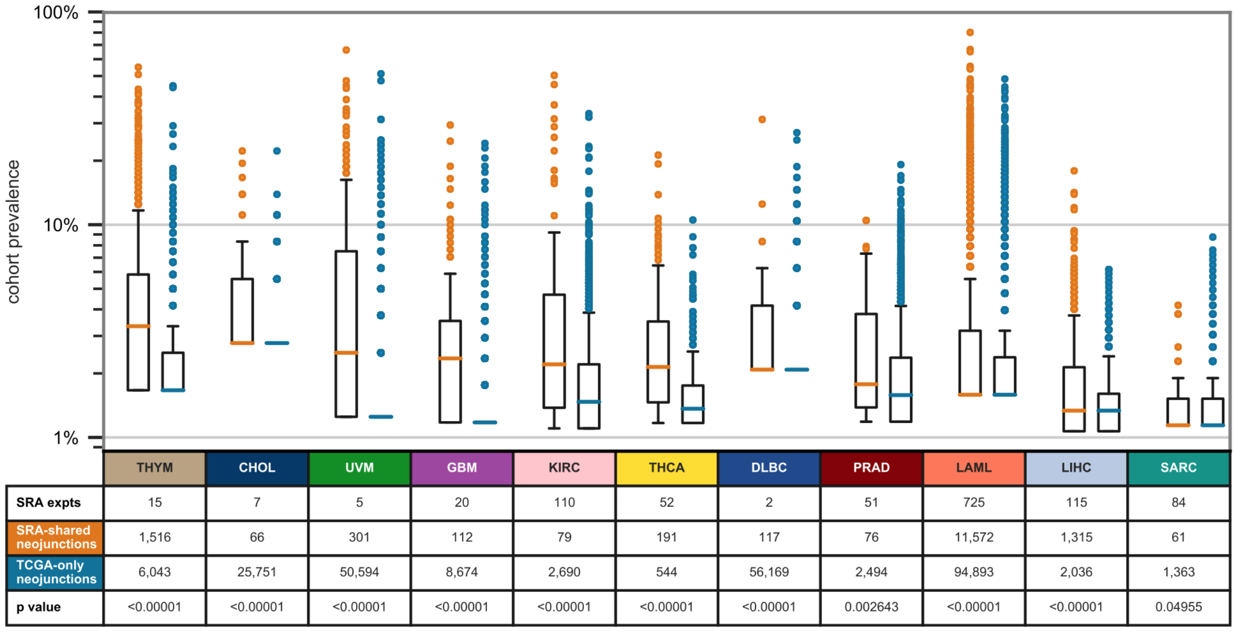

	THYM	CHOL	UVM	GBM	KIRC	THCA	DLBC	PRAD	LAML	LIHC	SARC
**SRA expts**	15	7	5	20	110	52	2	51	725	115	84
**SRA-shared neojunctions**	1,516	66	301	112	79	191	117	76	11,572	1,315	61
**TCGA-only neojunctions**	6,043	25,751	50,594	8,674	2,690	544	56,169	2,494	94,893	2,036	1,363
**p value**	<0.00001	<0.00001	<0.00001	<0.00001	<0.00001	<0.00001	<0.00001	0.002643	<0.00001	<0.00001	0.04955

Supplementary Figure S1: Distribution and prevalences of TCGA cancer sample junctions

(A) Log-scale scatterplot showing no relationship between junction burden (number of junctions per sample scaled by mapped read count) and tumor mutational burden. Each point represents one TCGA sample, colored based on cancer type (as in Figure 1B and Supplementary Figures S1B and S1C).

(B) Log-scale sorted strip plots representing the number of high-support junctions per sample for each of 33 TCGA cancer types where each point is a single TCGA tumor sample and the width of each strip is proportional to the size of the cancer type cohort (15). High support requires junction coverage within a sample to be equivalent to at least 5 out of 100 million 100-base pair reads. The upper panel counts junctions not found in GENCODE annotation or in tissue-matched normal GTEx or TCGA samples (Supplementary Table S1); the lower panel counts junctions not found in GENCODE annotation or in any core normal samples, differing from Figure 1B in that here, GENCODE-annotated junctions are also removed and the coverage filter is applied. The gray bars highlight TCGA cancer types with no or few tissue-matched normal samples (Supplementary Table S1); note that there are orders of magnitude fewer GENCODE-annotated junctions than junctions found in tissue-matched normal samples, partially explaining the high values for THYM, CESC, UVM, and DLBC in the upper panel.

(C) Log-scale scatter plot showing, for 33 TCGA cancer types, the number of junctions shared within each cancer-type cohort at each prevalence level, counting only junctions not found in any core normal samples. TCGA cancer type colors are as specified in Figures 1B, 2A, and S1B. Of interest with significant intra-cohort junction sharedness are, among others, ovarian carcinoma (tan), leukemia (pink), testicular germ cell tumors (red), and uveal melanoma (dark green).

(D) Log-scale histogram showing inter-cancer sharedness of junctions not found in core normal samples. Most junctions occur in only one cancer type, but many are shared between 2 or more.

(E) Log-scale box plots representing, for all TCGA cancer types individually, the prevalences within each cancer-type cohort of junctions occurring in at least 1% of cancer-type samples, separated into prevalences for (blue, left) junctions found in GTEx or TCGA tissue-matched normal samples (Supplementary Table S1); (green, center) junctions not found in tissue-matched normals but found in other core normal samples; and (yellow, right) junctions found in no core normal samples. Any junction found in multiple cancer types is represented by multiple data points, one for each cancer type in which it is found. Figure 1C condenses all data from this figure into one pan-cancer set.

(F) Cancer specific splicing junctions in patients with and without splicing associated gene mutations: log count of junctions not found in any core normal samples for each patient are plotted (top) across each cancer type in TCGA. Within each cancer, boxplots represent either patients with mutations in UniProt annotated splicing-related genes (left) or patients without any related mutations (right). Overall prevalence of relevant mutations in splicing-related for each cancer type are plotted below.

(G) Presents data in the same manner as E, but with comparison between patients with mutations only in genes described in the TCGA splicing paper (15) vs all other patients.

(H) Analysis of junction sharedness for patients within mutational cohorts: for each cancer, junctions not found in core normal samples are plotted based on sharedness across all patients in the cancer cohort (Y-axis) compared to deviation from expected sharedness (estimated odds ratio (log scale) based on Fisher's exact test) for only patients with mutations in UniProt annotated splicing-related genes (x-axis).

(I) Presents data in the same manner as G, but with deviation from expected sharedness calculated only for patients with mutations in genes described in the TCGA splicing paper (15). We found that no specific junctions show significantly enriched sharedness in patients carrying relevant mutations (Fisher's exact test FDR > 0.05 for all in both A and B), however there is a consistent shift towards higher than expected sharedness across the majority of cancers for patients carrying at least one of the mutations defined by the TCGA splicing paper (15).

(J) Comparison of TCGA-cohort prevalence of junctions occurring vs. not occurring in SRA cancer samples: log-scale box plots representing, for selected TCGA cancer types, the prevalences within each cancer-type cohort of junctions occurring in at least 1% of cancer-type samples, separated into prevalences for junctions (orange, left) found or (blue, right) not found in type-matched cancer sample(s) from the SRA. Selected TCGA cancer types are those for which cancer-matched SRA sample junctions are available from Snaptron (28) and at least 50 TCGA cancer junctions not found in core normal samples are present in the cancer-type matched SRA samples. Most junctions are TCGA-specific, but junctions that are also found in a type-matched SRA cancer cohort have on average higher TCGA-cohort prevalences.

(A)

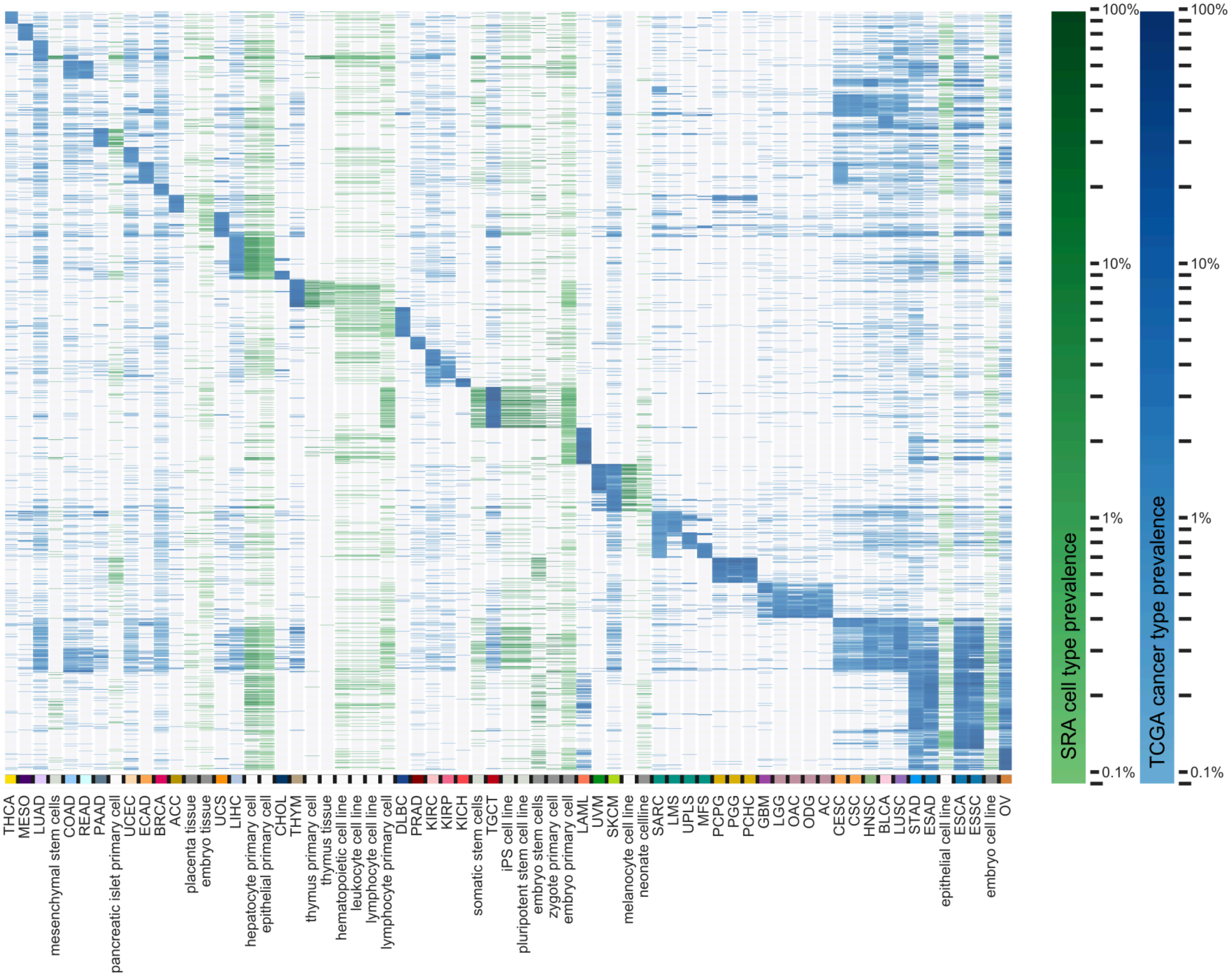
THCA
MESO
LUAD
mesenchymal stem cells
COAD
READ
PAAD
pancreatic islet primary cell
UCEC
ECAD
BRCA
ACC
placenta tissue
embryo tissue
UCS
LIHC
hepatocyte primary cell
epithelial primary cell
CHOL
THYM
thymus primary cell
thymus tissue
hematopoietic cell line
leukocyte cell line
lymphocyte cell line
lymphocyte primary cell
DLBC
PRAD
KIRC
KIRP
KICH
somatic stem cells
TGCT
iPS cell line
pluripotent stem cell line
embryo stem cells
zygote primary cell
embryo primary cell
LAML
UVM
SKCM
melanocyte cell line
neonate cellline
SARC
LMS
UPLS
MFS
PCPG
PGG
PCHC
GBM
LGG
OAC
ODG
AC
CESC
CSC
HNSC
BLCA
LUSC
STAD
ESAD
epithelial cell line
ESCA
ESSC
embryo cell line
OV
SRA cell type prevalence
100%
10%
1%
0.1%
TCGA cancer type prevalence
100%
10%
1%
0.1%

(B)

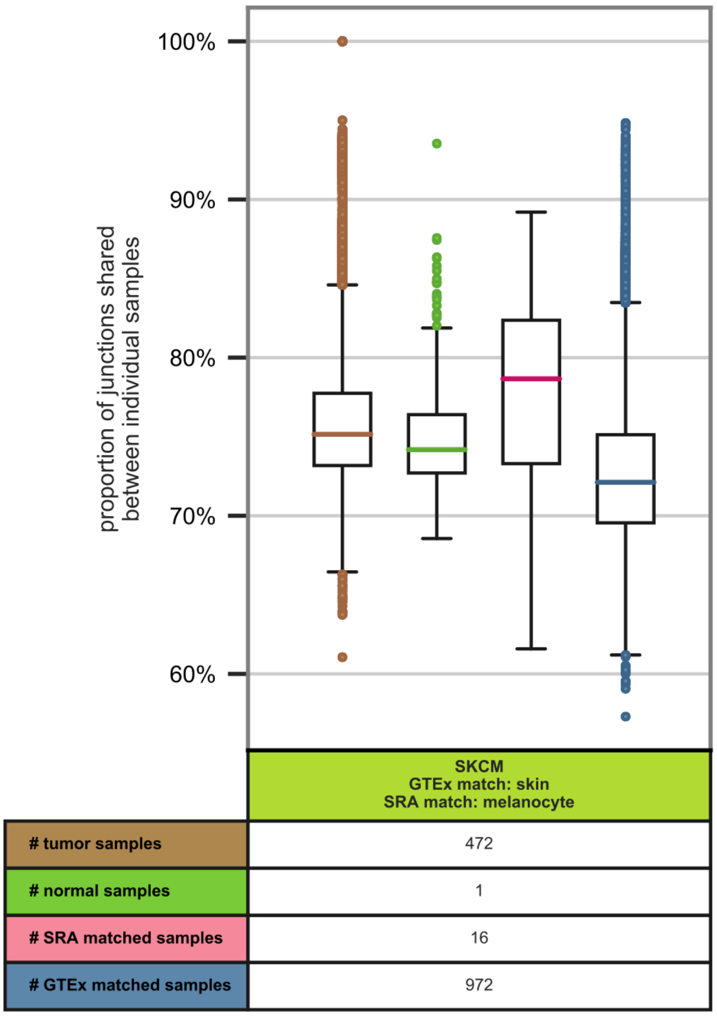

	SKCM<br>GTEx match: skin<br>SRA match: melanocyte
# tumor samples	472
# normal samples	1
# SRA matched samples	16
# GTEx matched samples	972

Supplementary Figure S2: Similarity of TCGA junctions and non-cancer SRA junctions

(A) Clustering by cohort prevalence of junctions not found in core normal samples: heatmap showing shared junction prevalences across all TCGA cancer type and associated histological subtypes with at least 20 samples. The clustered junctions are the 200 most prevalent junctions of each cancer type or subtype that are at least 1% prevalent in that subtype and are not found in any core normal samples but are found in at least one of the 22 non-cancer tissue and cell type SRA samples. Each heatmap row represents a junction's prevalence in each of the TCGA and SRA sample-type cohorts. The color bar beneath the plot shows SRA tissue and cell types colored according to their assigned categories (Supplementary Table S3), where white represents adult normal samples, light gray represents stem cell samples, and darker gray represents developmental samples.

(B) Sample-wise comparison of junctions from TCGA melanoma samples and select normal samples: boxplots showing the percent of junctions shared for every pairwise combination of TCGA melanoma tumor samples with (brown) TCGA melanoma tumor samples, (grass green) the single TCGA melanoma paired normal sample, (pink) SRA normal melanocyte samples (see Supplementary Tables S1 and S3), and (blue) GTEx normal skin samples. The percent of junctions shared between two samples is given by % shared = (set A & set B) / min(size(set A), size(set B)), where a set comprises all junctions identified in the single cancer or normal sample. TCGA melanoma cancer samples have on average a greater similarity of junctions to SRA normal melanocyte samples than to GTEx or TCGA bulk skin normal samples.

(A)

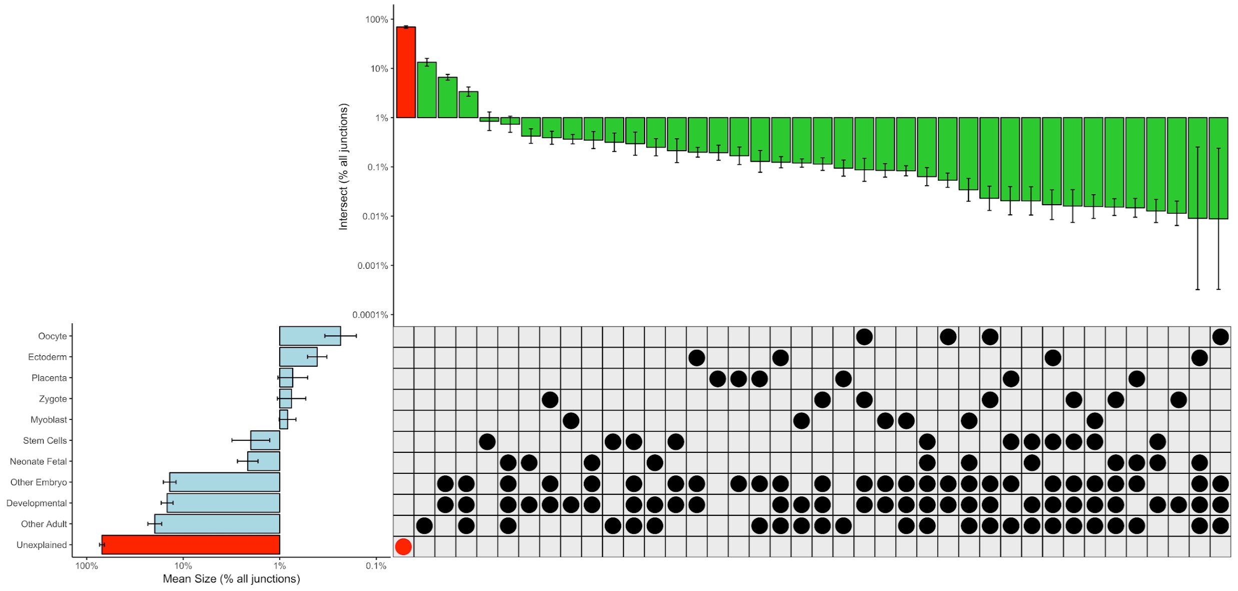

(B)

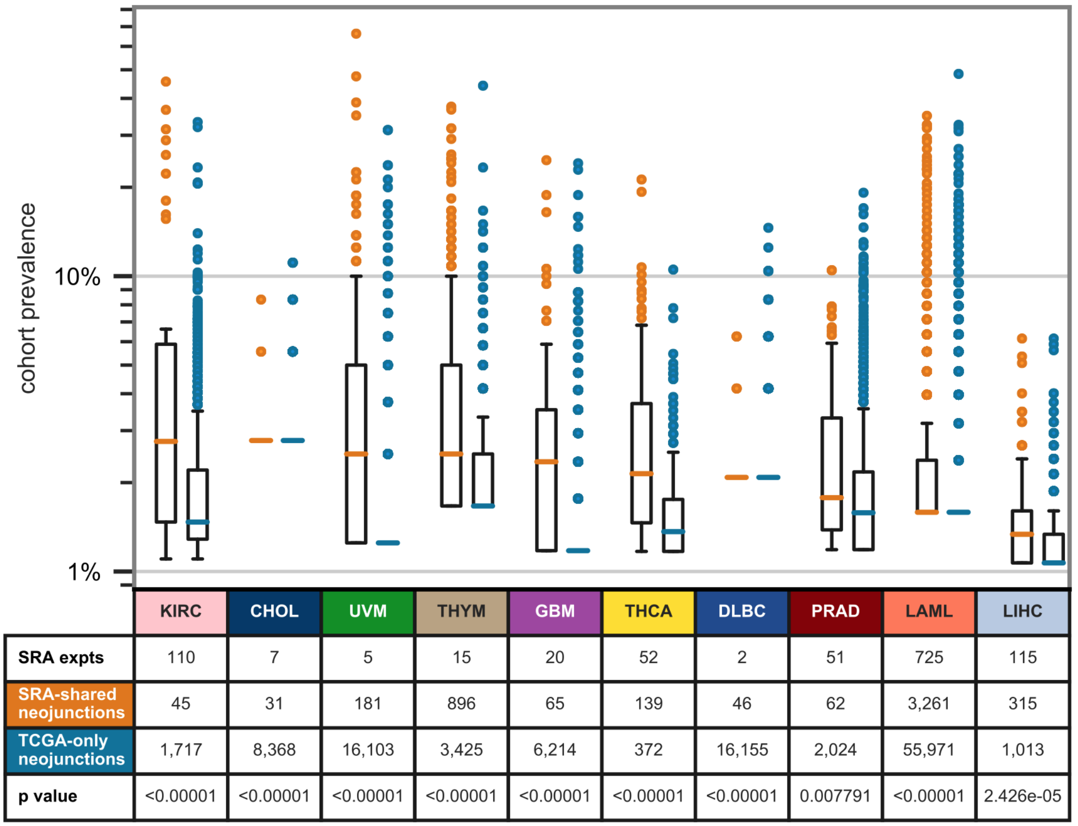

	KIRC	CHOL	UVM	THYM	GBM	THCA	DLBC	PRAD	LAML	LIHC
**SRA expts**	110	7	5	15	20	52	2	51	725	115
**SRA-shared neojunctions**	45	31	181	896	65	139	46	62	3,261	315
**TCGA-only neojunctions**	1,717	8,368	16,103	3,425	6,214	372	16,155	2,024	55,971	1,013
**p value**	<0.00001	<0.00001	<0.00001	<0.00001	<0.00001	<0.00001	<0.00001	0.007791	<0.00001	2.426e-05

(C)

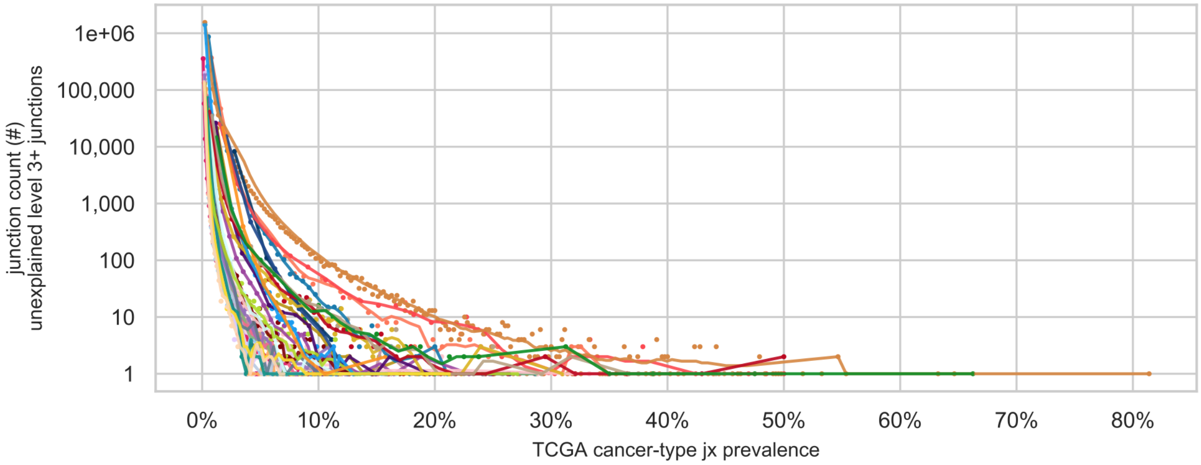

1e+06
100,000
10,000
1,000
100
10
1
junction count (#)
unexplained level 3+ junctions
0%
10%
20%
30%
40%
50%
60%
70%
80%
TCGA cancer-type jx prevalence

(D)

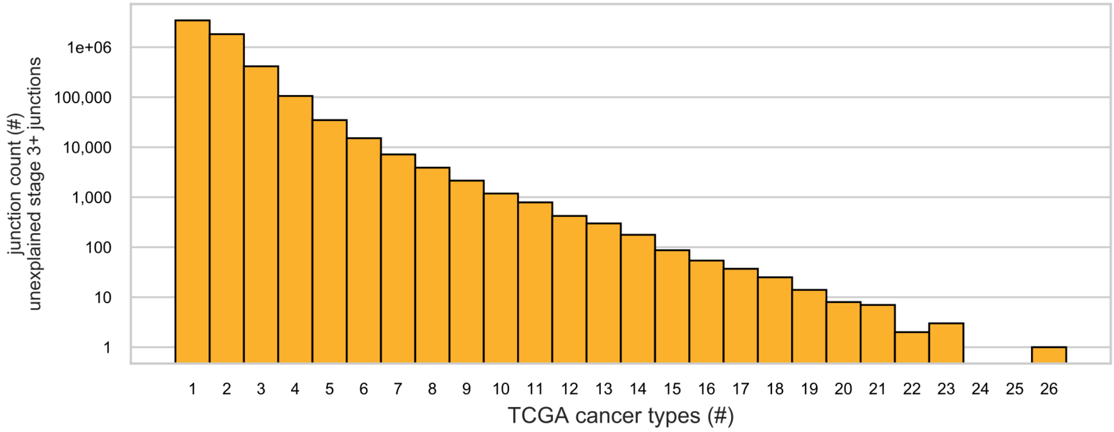

1e+06
100,000
10,000
1,000
100
10
1
junction count (#)
unexplained stage 3+ junctions
1 2 3 4 5 6 7 8 9 10 11 12 13 14 15 16 17 18 19 20 21 22 23 24 25 26
TCGA cancer types (#)

(E)

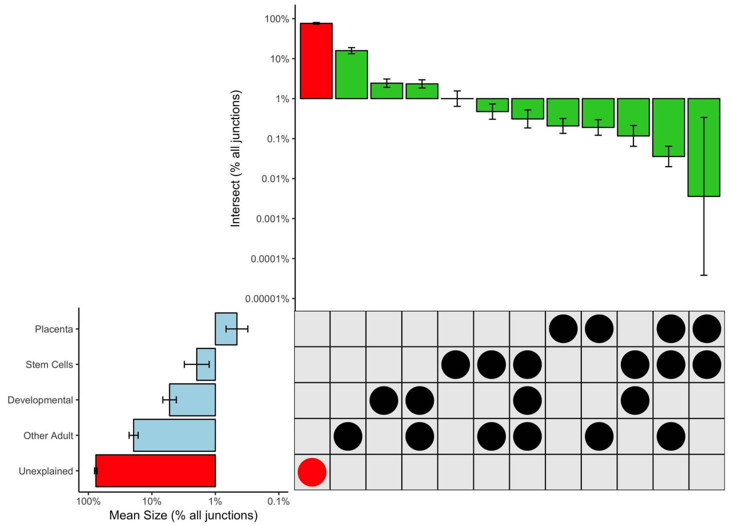

100%
10%
1%
0.1%
0.01%
0.001%
0.0001%
0.00001%
Intersect (% all junctions)
Placenta
Stem Cells
Developmental
Other Adult
Unexplained
100%
10%
1%
0.1%
Mean Size (% all junctions)

(F)

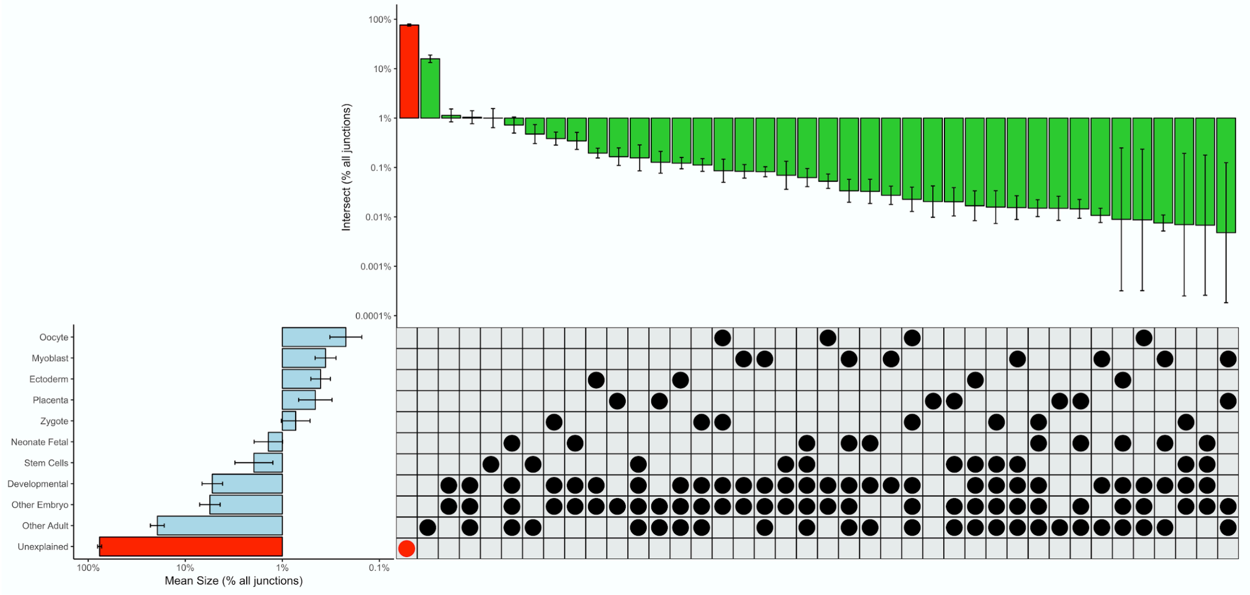
100%
10%
1%
0.1%
0.01%
0.001%
0.0001%
Intersect (% all junctions)
Oocyte
Myoblast
Ectoderm
Placenta
Zygote
Neonate Fetal
Stem Cells
Developmental
Other Embryo
Other Adult
Unexplained
100%
10%
1%
0.1%
Mean Size (% all junctions)

(G)

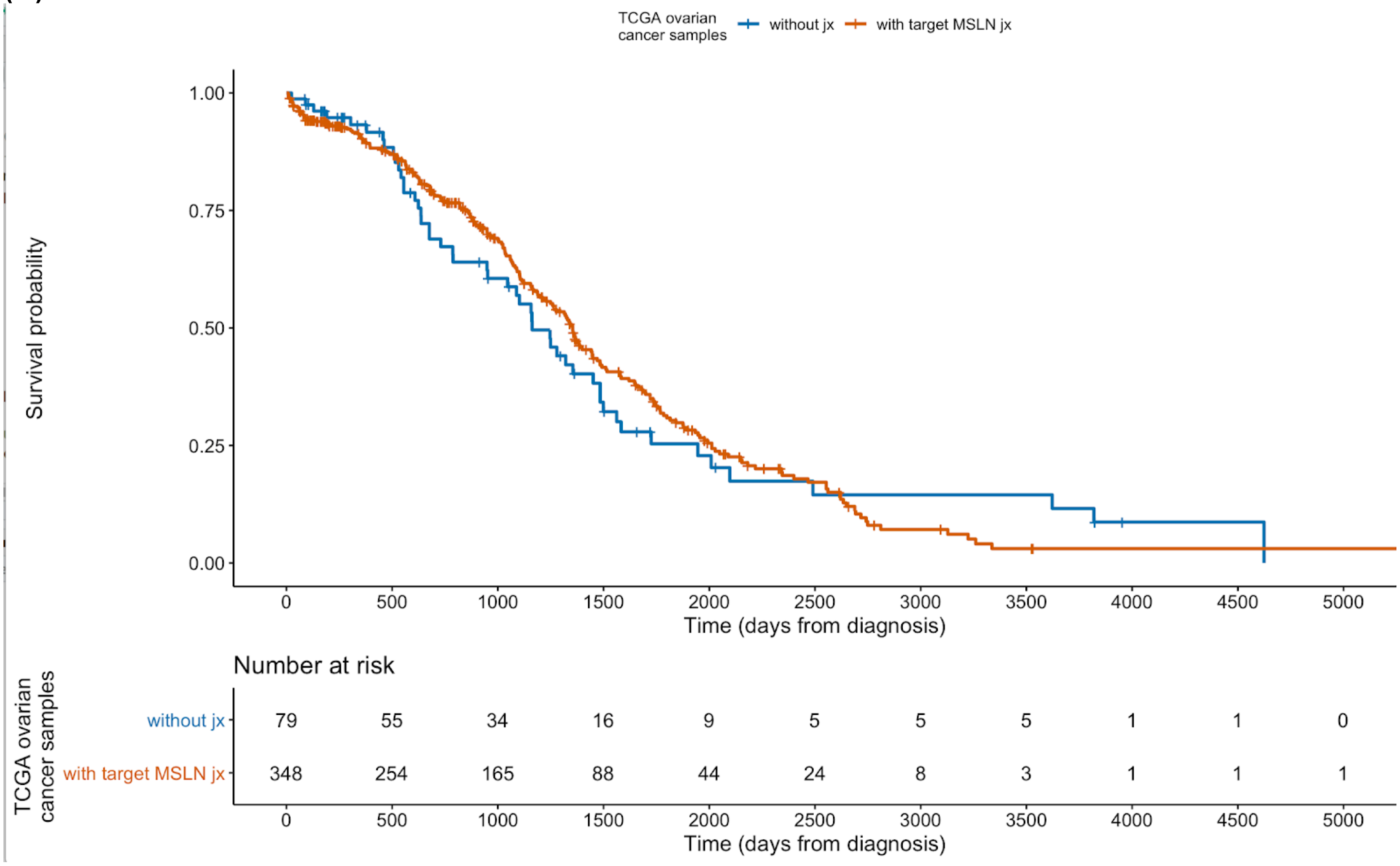
TCGA ovarian cancer samples
without jx
with target MSLN jx
Survival probability
1.00
0.75
0.50
0.25
0.00
0
500
1000
1500
2000
2500
3000
3500
4000
4500
5000
Time (days from diagnosis)
Number at risk
TCGA ovarian cancer samples
without jx
79 55 34 16 9 5 5 5 1 1 0
with target MSLN jx
348 254 165 88 44 24 8 3 1 1 1
Time (days from diagnosis)

Supplementary Figure S3: Distribution of junctions not found in core normal samples and unexplained junctions
(A) Expanded junction set assignments in normal tissue and cell type categories from the Sequence Read Archive, across cancers: upset-style plot with bar plots showing junction abundances across major sets and subsets (left) and set overlaps (top) across 33 cancers (error bars). Shown junctions are absent from all core normals. Unexplained junctions (red highlights) comprise junctions not present in any set categories studied (see also Figure 3A). The developmental set comprises human development-related junctions not present in the category placenta. Scale is log10 of percent of junctions not found in core normals, calculated for each cancer.
(B) Analysis of inter- and intra-cancer sharedness of stage-3+ “unexplained” junctions: log-scale box plots as in Supplementary Figure S1J but including only stage-3+ unexplained junctions not found in core normal samples or selected SRA normal adult, developmental, or stem cell samples (Table 1). Plots are presented for TCGA cancer types for which cancer-matched SRA sample junctions are available from Snaptron (28) and at least 30 unexplained junctions are present in the cancer-type matched SRA samples. Prevalences are given within each cancer-type cohort of junctions occurring in at least 0.5% of cancer-type samples, separated into prevalences for junctions (orange, left) found or (blue, right) not found in type-matched cancer sample(s) from the SRA. For all cancer types except DLBC, most junctions are TCGA-specific, but junctions that are also found in a type-matched SRA cancer cohort have on average higher TCGA-cohort prevalences.
(C) Log-scale scatter plot showing, for 33 TCGA cancer types, the number of level-3+ unexplained junctions shared within each cancer-type cohort at each prevalence level as in Figure Supplementary S1C. TCGA cancer type colors are as specified in Figures 1B, 2A, and S1B. Again, among others, ovarian carcinoma (tan), leukemia (pink), testicular germ cell tumors (red), and uveal melanoma (dark green) have significant intra-cohort junction sharedness.
(D) Log-scale histogram showing inter-cancer sharedness of stage-3+ unexplained junctions as in Supplementary Figure S1D. Again, most junctions occur in only one cancer type, but many are shared between 2 or more.
(E) Upset-style plot with bar plots showing junction abundances across major sets (left) and set overlaps (top) across 33 cancers (error bars); similar to Figure 3A, but presence in 2 samples across the SRA sample-type category is required for inclusion in a set. Shown junctions are absent from all core normals. Unexplained junctions (red highlights) comprise junctions not present in any set categories studied. The developmental set comprises human development-related junctions not present in the category placenta. Scale is log10 of percent of junctions not found in core normals, calculated for each cancer.
(F) Upset-style plot with bar plots showing junction abundances across major sets and subsets (left) and set overlaps (top) across 33 cancers (error bars); similar to Figure 3A, but presence in 2 samples across the SRA sample-type category is required for inclusion in a set. Shown junctions are absent from all core normals. Unexplained junctions (red highlights) comprise junctions not present in any set categories studied. The developmental set comprises human development-related junctions not present in the category placenta. Scale is log10 of percent of junctions not found in core normals, calculated for each cancer.
(G) Survival curve for patients with or without the target high-prevalence antisense MSLN junction of interest (chr16;766903;768491;-), censored at last registered follow-up appointment.

Supplementary Table S1: Sources and counts of tumor and tissue-matched normal samples

TCGA cancer type (Abbreviation: # tumor samples)	# of TCGA paired normal samples	GTEx matched tissue(s) (# of normal samples)	# TCGA tumor sample junctions not in tissue-matched normal (avg #/sample)	# unique TCGA cancer-type junctions not in tissue-matched normals	Additional matched normals used: SRA cell type of origin (# of samples)	Histological subtypes (Abbreviation: # of tumor samples)	SRA matched cancer: # of SRA samples
Acute Myeloid Leukemia (LAML: 126)	0	Blood (595)	2,836,278 (22,510/sample)	2,264,159	NA	NA	Acute myeloid leukemia: 725
Bladder Urothelial Carcinoma (BLCA: 414)	19	Bladder (11)	5,627,108 (13,592/sample)	2,227,034	NA	NA	NA
Brain Lower Grade Glioma (LGG: 532)	48	Brain (1409)	1,608,421 (3,023/sample)	1,266,167	NA	Astrocytoma (AC: 196); Oligoastrocytoma (OAC: 135); Oligodendroglioma (ODG: 200)	NA
Breast Invasive Carcinoma (BRCA: 1134)	112	Breast (218)	6,287,963 (5,545/sample)	3,480,255	NA	NA	NA
Cervical Squamous Cell Carcinoma & Endocervical Adenocarcinoma (CESC: 306)	3	Cervix Uteri (11)	14,086,434 (46,034 /sample)	2,263,326	NA	Cervical Adenosquamous (CASC: 6); Cervical squamous cell carcinoma (CSC: 253); Endocervical adenocarcinoma (ECAD: 47)	Cervical Carcinoma: 23
Colon Adenocarcinoma (COAD: 505)	41	Colon (376)	1,754,895 (3,475/sample)	1,268,454	NA	NA	NA
Esophageal Carcinoma (ESCA: 185)	13	Esophagus (788)	6,588,463 (35,613/sample)	4,827,828	NA	Esophagus Adenocarcinoma (ESAD: 89); Esophagus Squamous Cell Carcinoma (ESSC: 96)	NA
Glioblastoma Multiforme (GBM: 175)	5	Brain (1409)	922,811 (5,428/sample)	796,026	NA	NA	Glioblastoma multiforme: 20
Kidney Chromophobe (KICH: 66)	25	Kidney (36)	667,651 (10,116/sample)	471,050	NA	NA	NA
Kidney Renal Clear Cell Carcinoma (KIRC: 544)	72	Kidney (36)	4,593,062 (8,443/sample)	2,452,391	NA	NA	Renal Cell Carcinoma: 110
Kidney Renal Papillary Cell Carcinoma (KIRP: 291)	32	Kidney (36)	2,066,360 (7,102/sample)	1,272,647	NA	NA	NA
Liver Hepatocellular Carcinoma (LIHC: 374)	50	Liver (136)	1,968,456 (5,263/sample)	1,187,750	Hepatocyte cell line (7); Hepatocyte primary cells (77)	NA	Hepatocellular carcinoma: 115

Lung Adenocarcinoma (LUAD: 542)	59	Lung (374)	2,827,233 (5,216/sample)	1,885,643	NA	NA	Lung adenocarcinoma: 35
Lung Squamous Cell Carcinoma (LUSC: 504)	51	Lung (374)	3,575,668 (7,095/sample)	1,888,833	NA	NA	NA
Ovarian Serous Cystadenocarcinoma (OV: 430)	0	Ovary (108), Fallopian Tube (7)	30,141,239 (70,096/sample)	11,483,211	NA	NA	NA
Pancreatic Adenocarcinoma (PAAD: 179)	4	Pancreas (197)	1,315,090 (7,347/sample)	830,468	NA	NA	NA
Prostate Adenocarcinoma (PRAD: 506)	52	Prostate (119)	2,476,764 (4,895/sample)	1,643,727	NA	NA	Prostate adenocarcinoma: 51
Skin Cutaneous Melanoma (SKCM: 472)	1	Skin (972)	1,935,123 (4,100/sample)	1,198,791	NA	NA	NA
Rectum Adenocarcinoma (READ: 167)	10	Colon (376)	625,914 (3,748/sample)	496,129	NA	NA	NA
Stomach Adenocarcinoma (STAD: 416)	37	Stomach (203)	12,378,838 (29,757/sample)	7,764,375	NA	NA	NA
Testicular Germ Cell Tumors (TGCT: 156)	0	Testis (203)	935,595 (5,997/sample)	573,824	NA	NA	Testicular cancer: 9
Thyroid Carcinoma (THCA: 513)	59	Thyroid (361)	1,783,492 (3,477/sample)	1,296,521	NA	NA	Thyroid carcinoma: 52
Uterine Carcinosarcoma (UCS: 57)	0	Uterus (90)	593,740 (10,416/sample)	413,469	NA	NA	NA
Uterine Corpus Endometrial Carcinoma (UCEC: 554)	35	Uterus (90)	3,542,334 (6,394/sample)	1,968,191	NA	NA	NA
Sarcoma (SARC: 263)	2	Adipose Tissue (620), Muscle (475)	1,739,153 (6,613/sample)	1,069,549	NA	Desmoid Tumor (DT: 2); Leiomyosarcoma (LMS: 106); Malignant Peripheral Nerve Sheath Tumors (MPNT: 10); Myxofibrosarcoma (MFS: 25); Synovial Sarcoma (SYNS: 10); Undifferentiated Pleomorphic Sarcoma (UPLS: 52)	Sarcoma: 84
Pheochromocytoma & Paraganglioma (PCPG: 184)	3	Adrenal Gland (159), Nerve (335)	1,168,368 (6,350/sample)	662,442	NA	Pheochromocytoma (PCHC: 151); Paraganglioma (PGG: 33)	NA

Adrenocortical Carcinoma (ACC: 79)	0	Adrenal Gland (159)	12,703,775 (160,807 /sample)	1,013,102	NA	NA	NA
Mesothelioma (MESO: 87)	0	Lung (374)	399,050 (4,587/sample)	317,630	NA	NA	NA
Head and Neck Squamous Cell Carcinoma (HNSC: 504)	44	Skin (972)	1,780,841 (3,533/sample)	1,262,383	NA	NA	NA
Cholangiocarcinoma (CHOL: 36)	9	Liver (136)	281,418 (7,817/sample)	220,929	NA	NA	Cholangio-carcinoma: 7
Thymoma (THYM: 120)	2	NA	4,228,476 (35,237 /sample)	1,206,043	Thymus primary cell (20); Thymus tissue (119)	NA	Thymoma: 15
Lymphoid Neoplasm Diffuse Large B-cell Lymphoma (DLBC: 48)	0	NA	No match: 7,722,928 (160,894 /sample)	849,592	NA	NA	Diffuse Large B-cell Lymphoma: 2
Uveal Melanoma (UVM: 80)	0	NA	No match: 13,315,153 (166,439/sample)	899,967	Melanocyte cell line (22); Melanocyte primary cell (8)	NA	Uveal Melanoma: 5
NA	NA	Heart (489)	NA	NA	NA	NA	NA
NA	NA	Pituitary (124)	NA	NA	NA	NA	NA
NA	NA	Salivary Gland (70)	NA	NA	NA	NA	NA
NA	NA	Small Intestine (104)	NA	NA	NA	NA	NA
NA	NA	Spleen (118)	NA	NA	NA	NA	NA
NA	NA	Vagina (97)	NA	NA	NA	NA	NA
NA	NA	Blood vessel (750)	NA	NA	NA	NA	NA

Supplementary Table S2: Percent of junctions not found in core normal samples, averaged across cancer types

SRA category:		Adult	Developmental	Stem Cells	Unexplained
Number of samples per SRA category required for set membership	1	26.5%	15.4%	2.7%	64.9%
	2	19.6%	5.92%	2.2%	76.4%

Supplementary Table S3: Selection of additional normal tissue and cell types analyzed

SRA cell or tissue type	Sample types (# of samples)	Assigned category > subcategory (if applicable)
Aorta	tissue (266)	adult
Astrocyte	cell line (4), primary cells (83)	adult
Biliary Tree	combined group of tissue (4 samples) and stem cells (3 samples) (7)	adult
Bone	cell line (20), tissue (58)	adult
Ectoderm	cell line (17)	developmental > embryonic
Embryo	cell line (929), primary cells (904), stem cells (34), tissue (989)	developmental > embryonic
Epithelial Cell	cell line (853), primary cell (621)	adult
	stem cells (57)	stem cells
Eye	cell line (42), primary cell (4), tissue (53)	adult
Fallopian Tube	tissue (13)	adult
Fibroblast	cell line (1660), primary cell (351)	adult
	stem cells (9)	stem cells
Glial Cell	cell line (10), primary cells (136)	adult
Hematopoietic Cell	cell line (603), primary cell (2679)	adult
	stem cells (18)	stem cells
Hepatocyte	cell line (7), primary cell (77)	adult
Induced Pluripotent Stem Cell	cell line (139)	stem cells
Pancreatic Islet	cell line (3), primary cell (285)	adult
Leukocyte	cell line (370), primary cell (2178)	adult
Lymphocyte	cell line (255), primary cell (1073)	adult
Macrophage	cell line (19), primary cell (130)	adult
Melanocyte	cell line (22), primary cell (8)	adult
Mesenchymal Stem Cell	stem cells (65)	stem cells
Mesenchyme	stem cells (19)	stem cells
Mesothelium	cell line (4)	adult

Myeloid Cell	cell line (29), primary cell (794)	adult
Myoblast	cell line (7), primary cell (402)	developmental > embryonic
Neonate	cell line (250), primary cell (105), tissue (21)	developmental > fetal
Oligodendrocyte	primary cell (37)	adult
Oocyte	primary cell (11)	developmental > oocyte
Placenta	tissue (264)	developmental > placental
Platelet	primary cell (6)	adult
Pluripotent Stem Cell	cell line (139), stem cells (6)	stem cells
Somatic Stem Cell	stem cells (86)	stem cells
Thymus	primary cell (20), tissue (119)	adult
Zygote	primary cell (27)	developmental > zygote

Supplementary Table S4: Unexplained junctions occurring in >10% of samples of multiple TCGA cancer types

Unexplained stage 3+ junction (chr; left splice site; right splice site; strand)	Cancer count	Cancer type: % of cancer-type samples containing the junction
chr18;49501731;49524503;-	3	Esophageal_Carcinoma: 21.0811%; Head_and_Neck_Squamous_Cell_Carcinoma: 28.7698%; Lung_Squamous_Cell_Carcinoma: 13.0952%
chr19;58392528;58393026;+	3	Esophageal_Carcinoma: 11.3514%; Ovarian_Serous_Cystadenocarcinoma: 19.3023%; Stomach_Adenocarcinoma: 12.9808%
chr11;2129478;2395551;+	2	Adrenocortical_Carcinoma: 13.9241%; Uterine_Carcinosarcoma: 10.5263%
chr11;22900551;23057663;-	2	Skin_Cutaneous_Melanoma: 11.4407%; Uveal_Melanoma: 12.5%
chr12;15569284;15578851;+	2	Colon_Adenocarcinoma: 15.4455%; Rectum_Adenocarcinoma: 16.1677%
chr12;15579385;15580037;+	2	Colon_Adenocarcinoma: 16.6337%; Rectum_Adenocarcinoma: 16.7665%
chr14;100376176;100734652;+	2	Adrenocortical_Carcinoma: 17.7215%; Pheochromocytoma_and_Paraganglioma: 22.2826%
chr18;49524635;49578176;-	2	Esophageal_Carcinoma: 15.6757%; Head_and_Neck_Squamous_Cell_Carcinoma: 18.6508%
chr19;3976571;3982811;-	2	Acute_Myeloid_Leukemia: 13.4921%; Ovarian_Serous_Cystadenocarcinoma: 10.4651%
chr19;41683816;41771127;-	2	Esophageal_Carcinoma: 14.5946%; Stomach_Adenocarcinoma: 15.8654%
chr19;41709948;41718251;+	2	Esophageal_Carcinoma: 11.3514%; Stomach_Adenocarcinoma: 10.8173%
chr21;16971056;16971076;+	2	Esophageal_Carcinoma: 13.5135%; Ovarian_Serous_Cystadenocarcinoma: 18.1395%
chr6;116004903;116119064;+	2	Esophageal_Carcinoma: 11.8919%; Stomach_Adenocarcinoma: 11.2981%
chr6;116004910;116119071;+	2	Esophageal_Carcinoma: 11.8919%; Stomach_Adenocarcinoma: 11.2981%
chr6;31944795;31944981;+	2	Ovarian_Serous_Cystadenocarcinoma: 19.5349%; Stomach_Adenocarcinoma: 11.7788%
chrX;4542633;4895036;+	2	Esophageal_Carcinoma: 10.8108%; Stomach_Adenocarcinoma: 11.0577%

Supplementary Table S5: Junction counts and proportion of antisense junctions for all TCGA cancer types

TCGA cancer type	Core normals		Other adult non-cancer		Developmental		Stem cell		Unexplained	
	junction count	antisense prevalence	junction count	antisense prevalence	junction count	antisense prevalence	junction count	antisense prevalence	junction count	antisense ratio
Acute Myeloid Leukemia	1,745,361	27%	183,003	46%	46,261	48%	4,580	45%	427,768	47%
Adrenocortical Carcinoma	941,404	20%	7,830	39%	2,369	38%	326	33%	22,053	44%
Bladder Urothelial Carcinoma	2,401,749	24%	58,874	35%	17,652	31%	3,348	26%	155,124	42%
Brain Lower Grade Glioma	2,847,326	26%	64,093	40%	19,716	38%	3,129	32%	165,091	41%
Breast Invasive Carcinoma	4,013,058	27%	170,106	36%	44,855	36%	7,520	30%	441,104	37%
Cervical Squamous Cell Carcinoma and Endocervical Adenocarcinoma	2,148,683	24%	45,625	36%	12,353	35%	2,361	27%	111,091	42%
Cholangiocarcinoma	754,166	15%	3,790	33%	900	33%	168	24%	8,399	37%
Colon Adenocarcinoma	2,228,830	25%	64,988	37%	19,894	31%	2,841	32%	155,589	42%
Esophageal Carcinoma	3,939,154	29%	349,351	43%	113,466	45%	13,813	38%	1,039,014	46%
Glioblastoma Multiforme	2,368,563	25%	35,154	36%	12,209	35%	2,024	26%	75,111	37%
Head and Neck Squamous Cell Carcinoma	2,571,867	24%	72,107	35%	19,318	34%	4,210	27%	177,027	40%
Kidney Chromophobe	1,042,201	22%	10,778	42%	3,081	37%	490	26%	28,961	42%
Kidney Renal Clear Cell Carcinoma	2,925,458	26%	93,435	38%	21,535	38%	3,160	32%	197,822	40%
Kidney Renal Papillary Cell Carcinoma	1,896,671	25%	33,239	39%	8,380	38%	1,255	33%	85,284	41%
Liver Hepatocellular Carcinoma	1,889,979	23%	43,871	36%	9,302	33%	1,486	27%	91,086	40%
Lung Adenocarcinoma	2,890,617	26%	95,291	37%	23,848	35%	4,387	30%	229,442	40%
Lung Squamous Cell Carcinoma	3,018,232	24%	92,301	33%	27,381	30%	5,604	22%	212,501	36%
Lymphoid Neoplasm Diffuse Large B cell Lymphoma	793,306	18%	9,851	30%	1,806	32%	277	30%	16,201	36%
Mesothelioma	1,150,647	20%	11,387	38%	3,060	39%	594	29%	27,214	43%
Ovarian Serous Cystadenocarcinoma	5,092,658	32%	542,738	46%	242,074	48%	24,032	41%	2,739,236	49%
Pancreatic Adenocarcinoma	1,593,779	22%	20,854	39%	5,087	41%	876	33%	49,429	44%
Pheochromocytoma and Paraganglioma	1,480,083	23%	19,085	40%	6,003	40%	832	35%	53,453	43%
Prostate Adenocarcinoma	2,423,109	26%	55,616	40%	15,372	40%	2,257	35%	159,220	43%
Rectum Adenocarcinoma	1,381,622	22%	21,823	37%	6,693	33%	945	28%	42,408	42%

Sarcoma	1,985,824	23%	31,671	35%	10,306	33%	1,699	28%	77,762	39%
Skin Cutaneous Melanoma	2,493,051	24%	62,444	36%	17,376	34%	2,973	27%	148,206	38%
Stomach Adenocarcinoma	4,844,395	31%	494,846	44%	172,922	46%	19,099	40%	1,787,745	47%
Testicular Germ Cell Tumors	1,627,329	19%	26,027	26%	12,435	23%	5,054	17%	49,836	31%
Thymoma	1,364,454	21%	19,938	32%	4,892	31%	1,057	26%	40,450	37%
Thyroid Carcinoma	2,501,295	27%	54,285	42%	14,124	42%	2,217	37%	147,977	44%
Uterine Carcinosarcoma	993,014	18%	8,062	35%	3,328	30%	601	29%	22,474	39%
Uterine Corpus Endometrial Carcinoma	2,499,341	26%	70,746	36%	20,596	35%	3,226	31%	155,889	41%
Uveal Melanoma	849,072	20%	7,281	40%	1,799	42%	276	35%	16,284	42%

Supplementary Table S6: Genes not currently cancer-associated with high novel junction burdens

Gene	Unexplained junctions (#)	Cancer types with >5% of samples containing unexplained junctions in this gene (#)
BCAM	212	1
MPO	121	1
MSLN	102	1
CLDN3	87	1
CHGA	69	1
LGALS3BP	36	2
SSPN	35	1
COL1A2	33	3
PKM	32	1
EEF2	31	2
SPON1	31	1
KRT7	31	1
ZNF503	30	1
C3	29	1
H1FX	28	1
MARCKSL1	27	1
CRIP2	26	1
AC007040.11	25	1
TNFAIP2	25	1
AGRN	25	2
